# Supplementary material for: Reconstruct high-resolution 3D genome structures for diverse cell-types using FLAMINGO
Source: Nat Commun. 2022 May 12;13:2645. doi: 10.1038/s41467-022-30270-2 (PMC9098643; doi:10.1038/s41467-022-30270-2)
Supplement: Supplementary file 1 — Supplementary Information [file 41467_2022_30270_MOESM1_ESM.pdf]

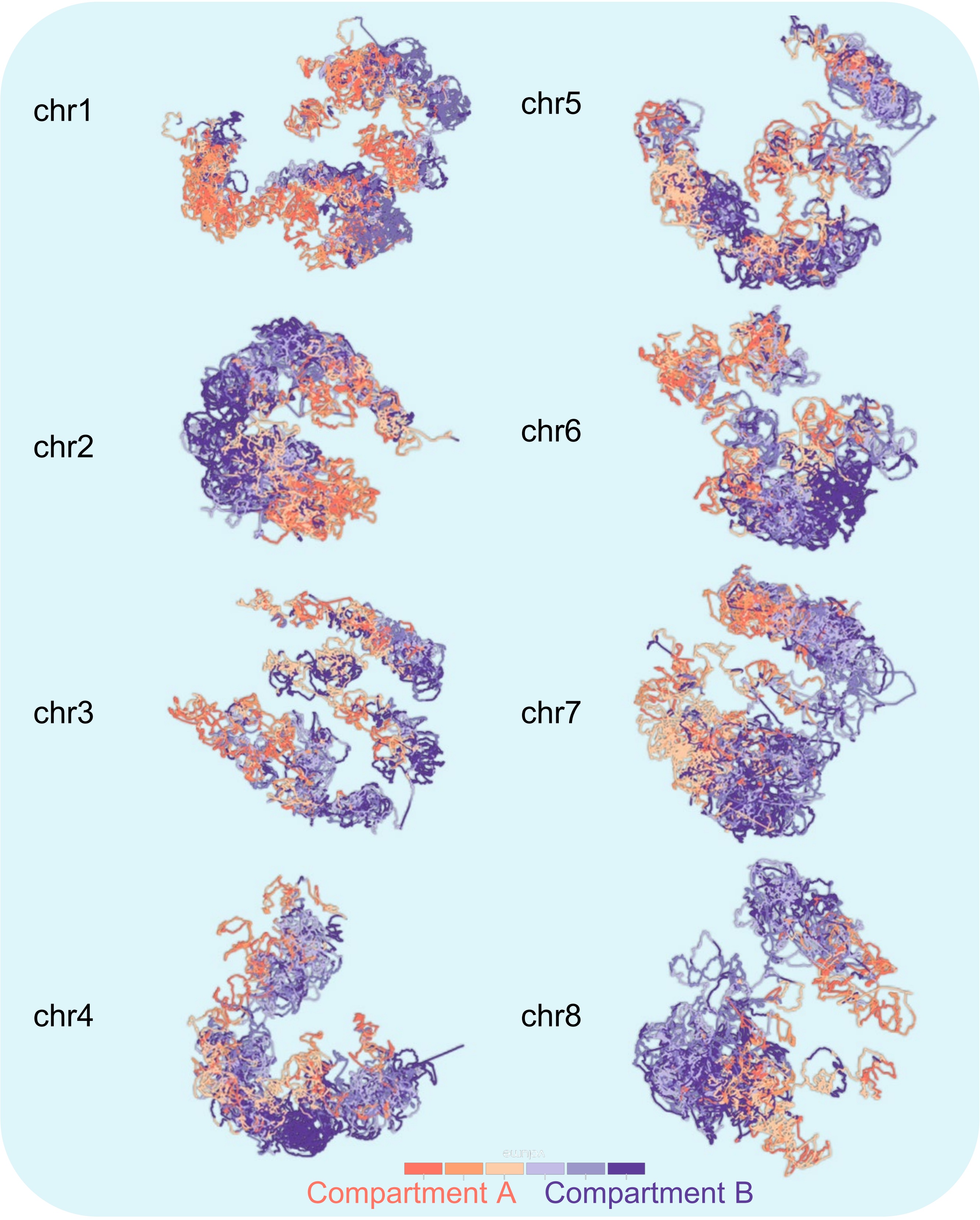

Supplementary Figure 1. 5kb-resolution 3D structures for 23 chromosomes predicted by FLAMINGO. Every chromosome (except chrX) is annotated by chromatin compartments (compartment A: orange; compartment B: blue). Consecutive TADs within each type of compartments are annotated by color gradients. The light orange/blue segments represent genomic regions that are not annotated as TADs. The compartment annotation for chromosome X is not available (grey). The left boxplot shows the distribution of predicted 3D distances for the anchors of significant Hi-C loops with CTCF motifs vs. the distribution of genomic-distance controlled random DNA fragment pairs (left, *n*=400, p-value = 2.78×10^{-5}, one-sided Wilcoxon test). The right boxplot further restricts the background pairs to be one Hi-C anchors with CTCF motifs and one random DNA fragments with genomic distance controlled (right, *n*=400, p-value = 5.21×10^{-4}, one-sided Wilcoxon test). The center lines of boxplots show the median, the upper and lower box limits show the 25th and 75th percentiles respectively. The whiskers extend up to 1.5 times the interquartile range away from the limits of the boxes. Outliers outside this range were removed from the figure. Source data are provided as a Source Data file.

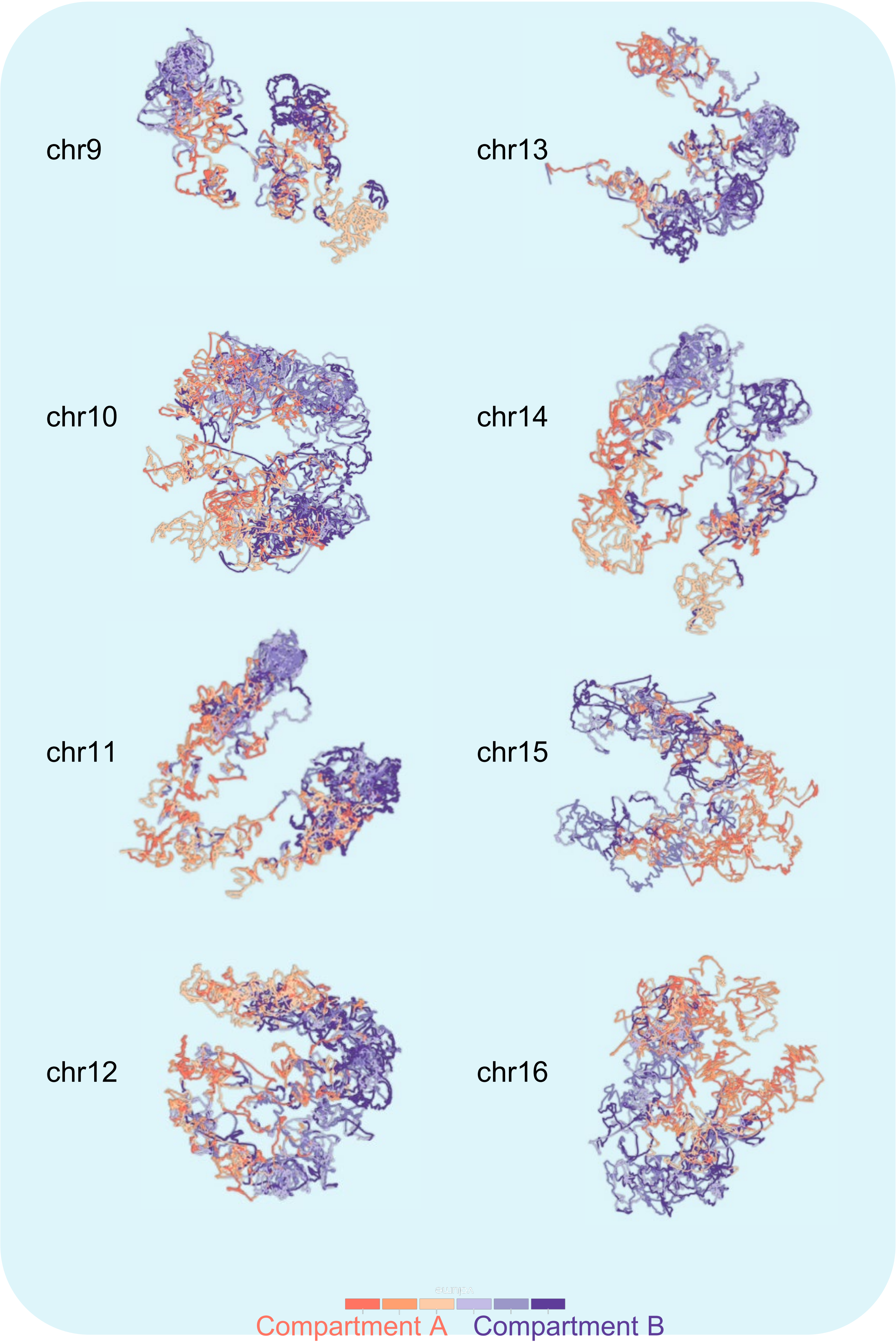
chr9
chr13
chr10
chr14
chr11
chr15
chr12
chr16
Compartment A
Compartment B

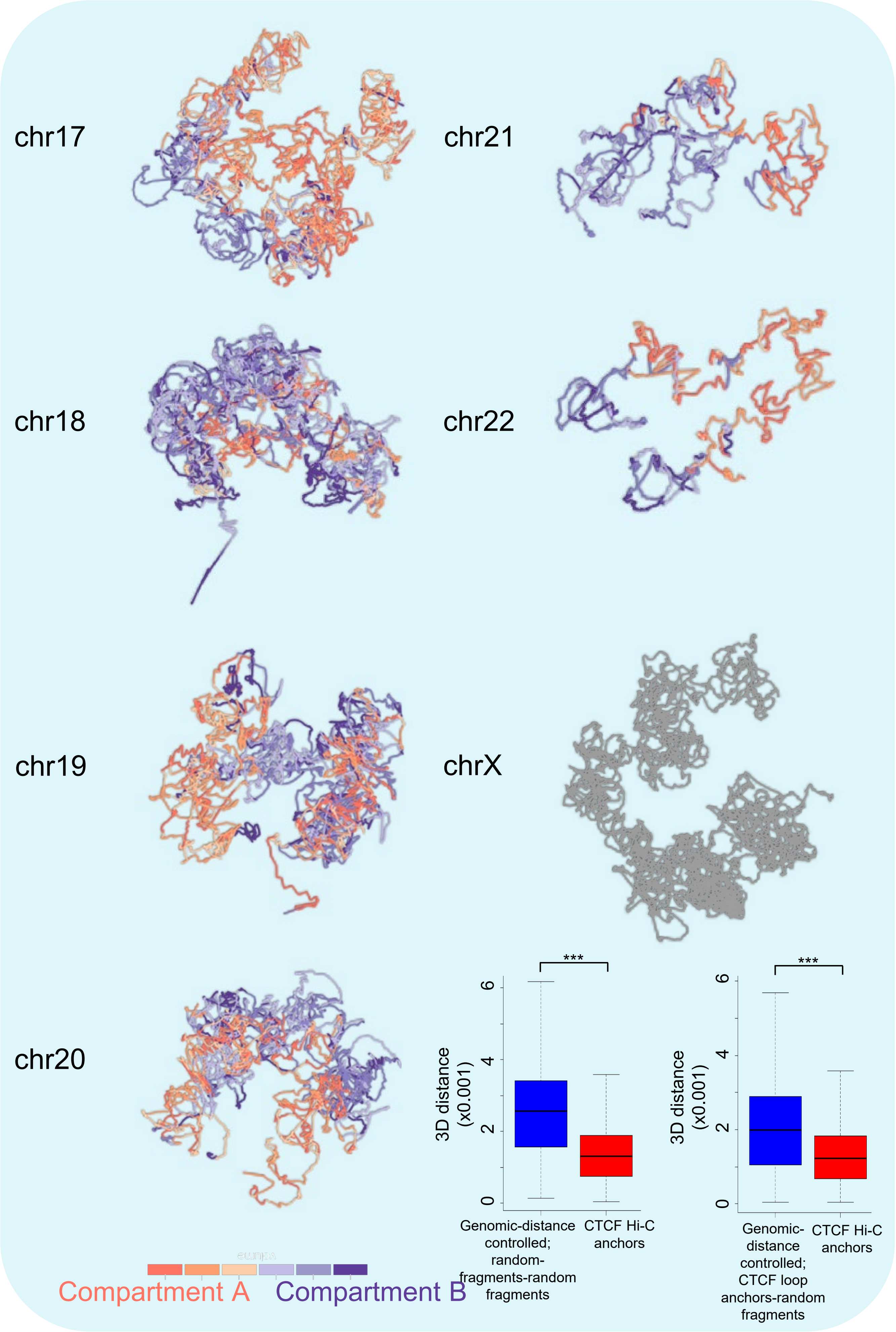
chr17
chr21
chr18
chr22
chr19
chrX
chr20
***
3D distance (x0.001)
0
2
4
6
Genomic-distance controlled; random-fragments-random fragments
CTCF Hi-C anchors
***
3D distance (x0.001)
0
2
4
6
Genomic-distance controlled; CTCF loop anchors-random fragments
CTCF Hi-C anchors
Compartment A
Compartment B

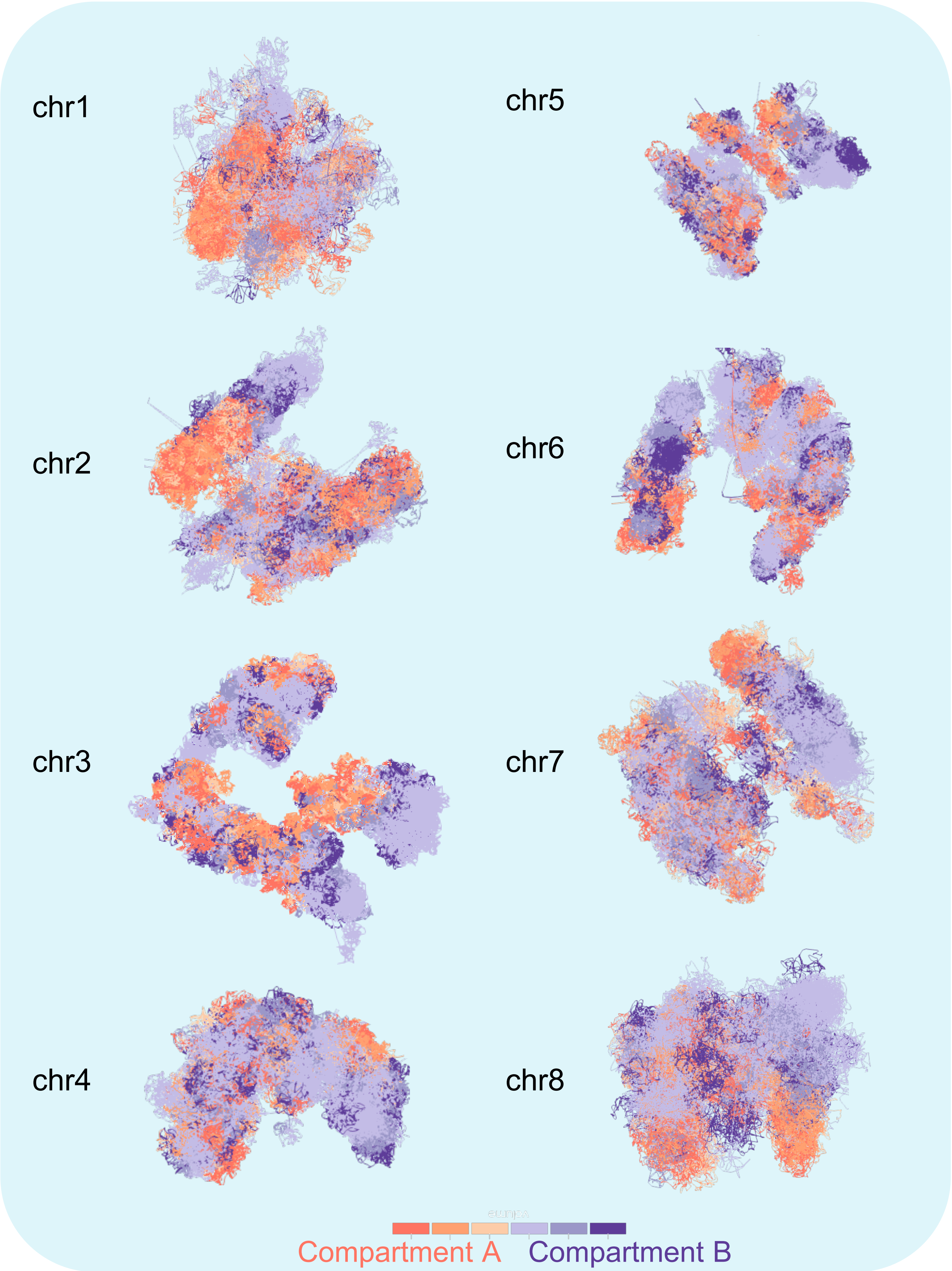

Supplementary Figure 2. The 3D structures predicted by FLAMINGO at 1kb-resolution for all 23 chromosomes. Every chromosome is annotated by chromatin compartments (compartment A: orange; compartment B: blue). Consecutive TADs within each type of compartments are annotated by color gradients. The light orange/blue segments represent genomic regions that are not annotated as TADs. The compartment annotation for chromosome X is not available (grey). The bar plot shows the performance evaluation of the predicted 3D structures at 1kb-resolution using all-points correlations and intra-domain correlations, compared with measured distances converted from Hi-C. The bar plot shows the average (mean) correlations across n=23 chromosomes and the error bars represent the standard deviations across n=23 chromosomes. Source data are provided as a Source Data file.

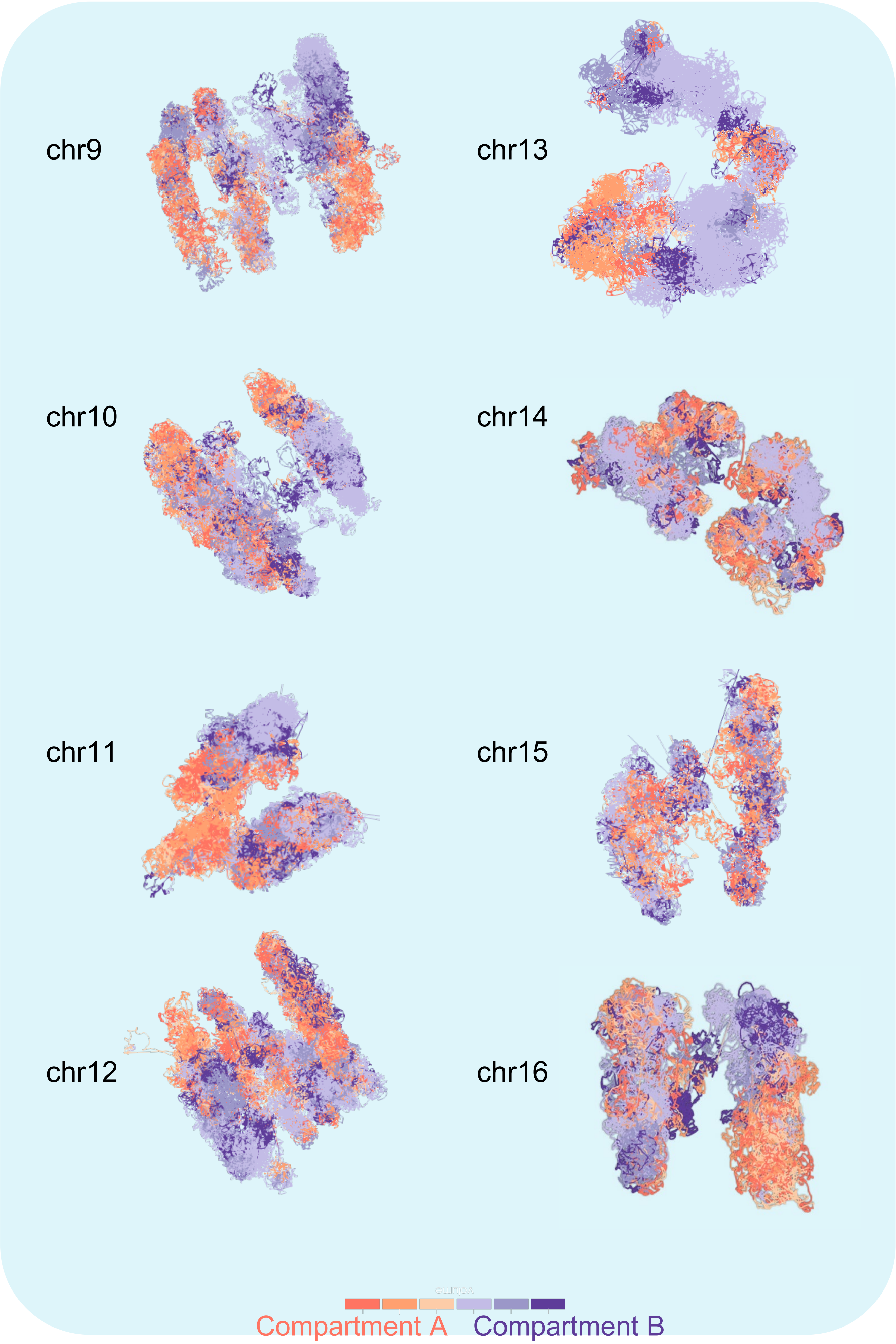
chr9
chr13
chr10
chr14
chr11
chr15
chr12
chr16
Compartment A
Compartment B

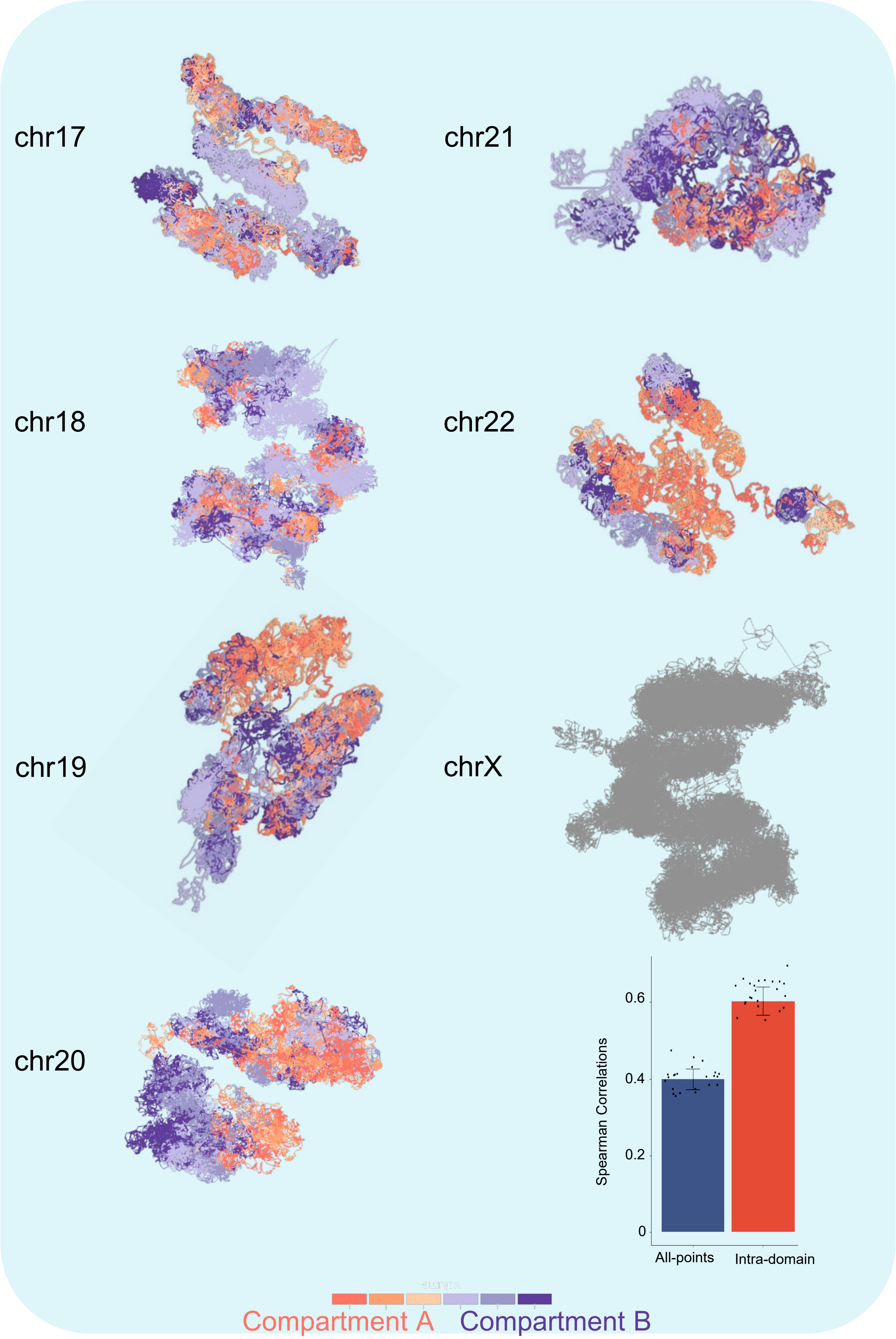
chr17
chr21
chr18
chr22
chr19
chrX
chr20
Spearman Correlations
0.6
0.4
0.2
0
All-points
Intra-domain
Compartment A
Compartment B

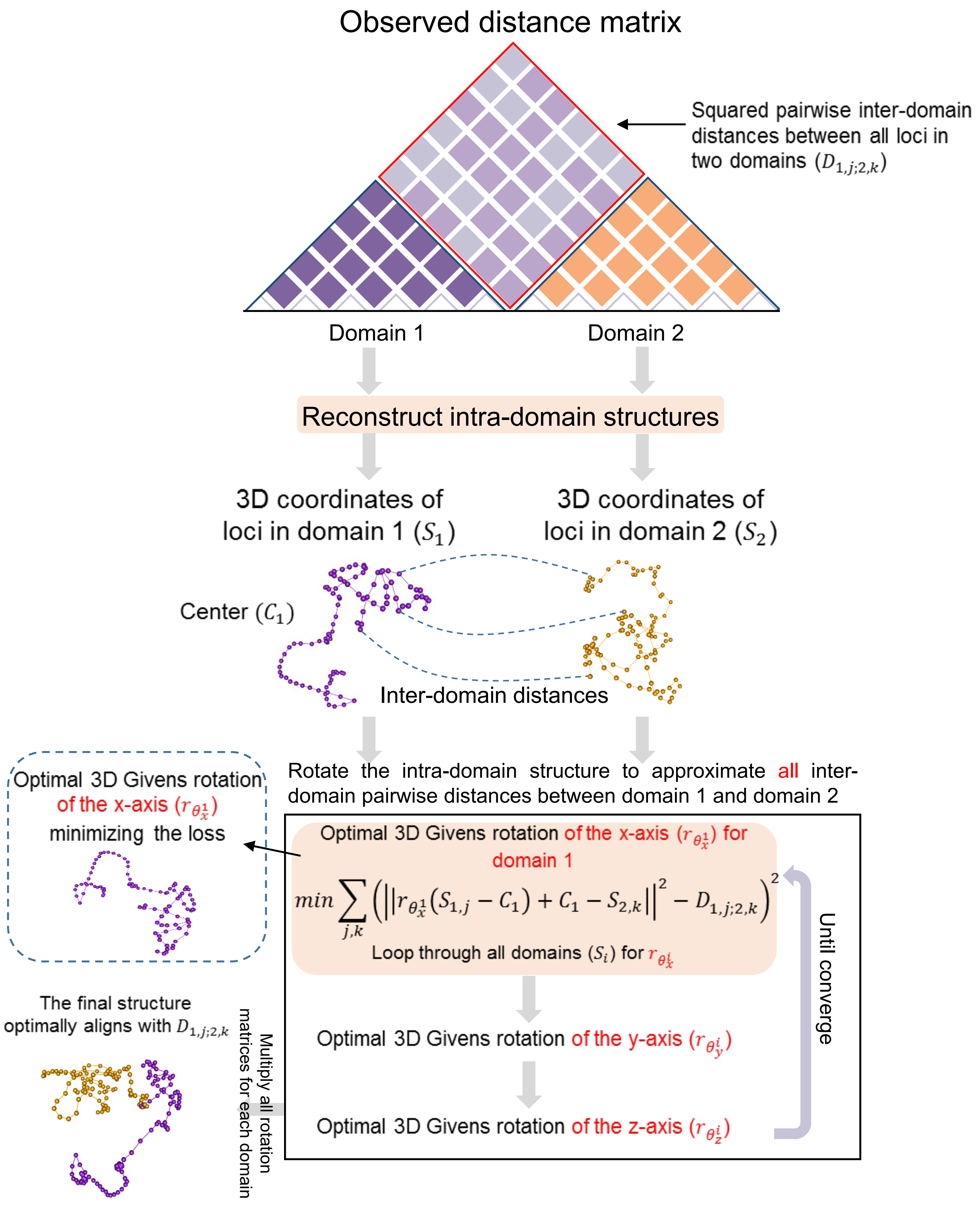

Supplementary Figure 3. Overview of the assembly algorithm of FLAMINGO. After FLAMINGO reconstructs the intra-domain structures along each chromosome, the adjacent domains, *e.g.* S_1 and S_2, are iteratively rotated to match with the observed inter-domain pairwise distances between 5kb DNA fragments from different domains (*i.e.* off-diagonal points in the matrix). FLAMINGO searches for the optimal 3D Givens rotations of the x-axis through adjacent domains, and then search for optimal Givens rotations of the y- and z-axis. Through iterations, FLAMINGO will identify the approximate optimal 3D rotations for each domain to minimize the differences between the reconstructed and observed inter-domain distances. Upon convergence, the optimally rotated domains are assembled to generate the final chromosome structures.

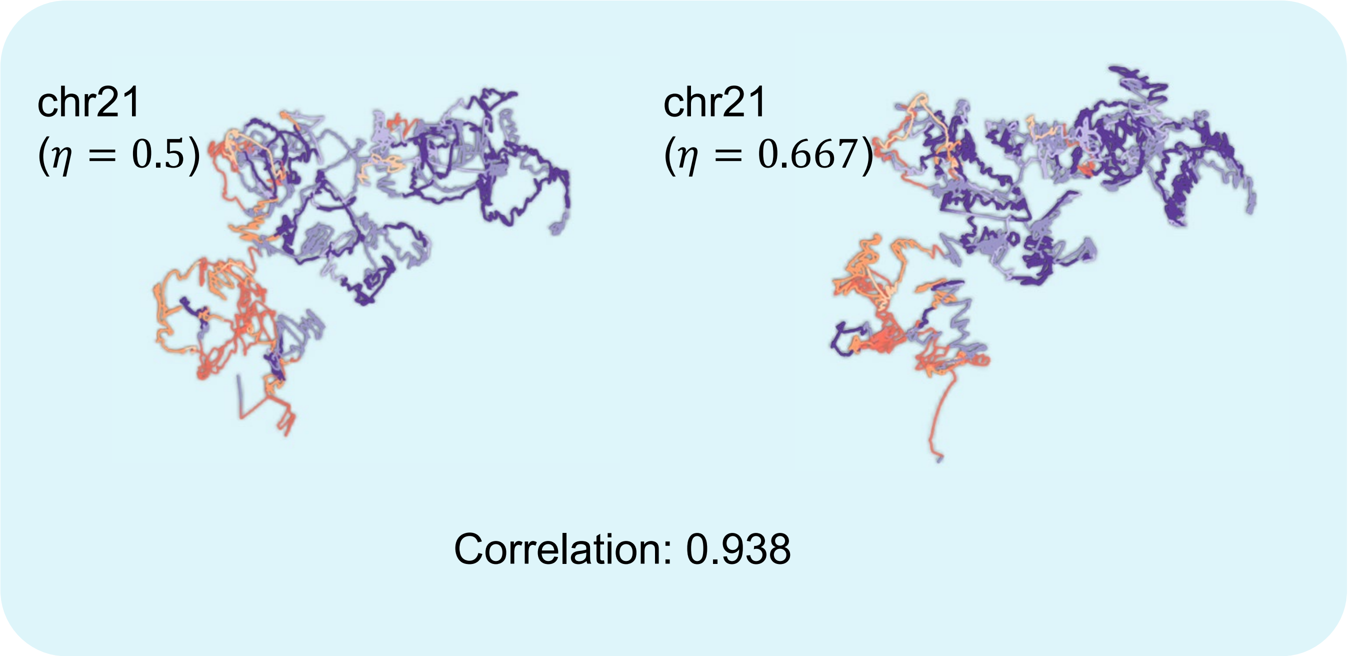

Supplementary Figure 4. Reconstructed 3D structure of chromosome 21 based on the observed distance matrix generated by different conversion factors from Hi-C interaction frequency. Two conversion factors ($\eta = 0.5$ and $\eta = 0.667$ for the squared distance matrix, which correspond to $\eta/2 = 0.25$ and $\eta/2 = 0.333$ as estimated by previous studies) are tested. The robustness of FLAMINGO is quantified by the high Spearman correlation between the two predicted structures.

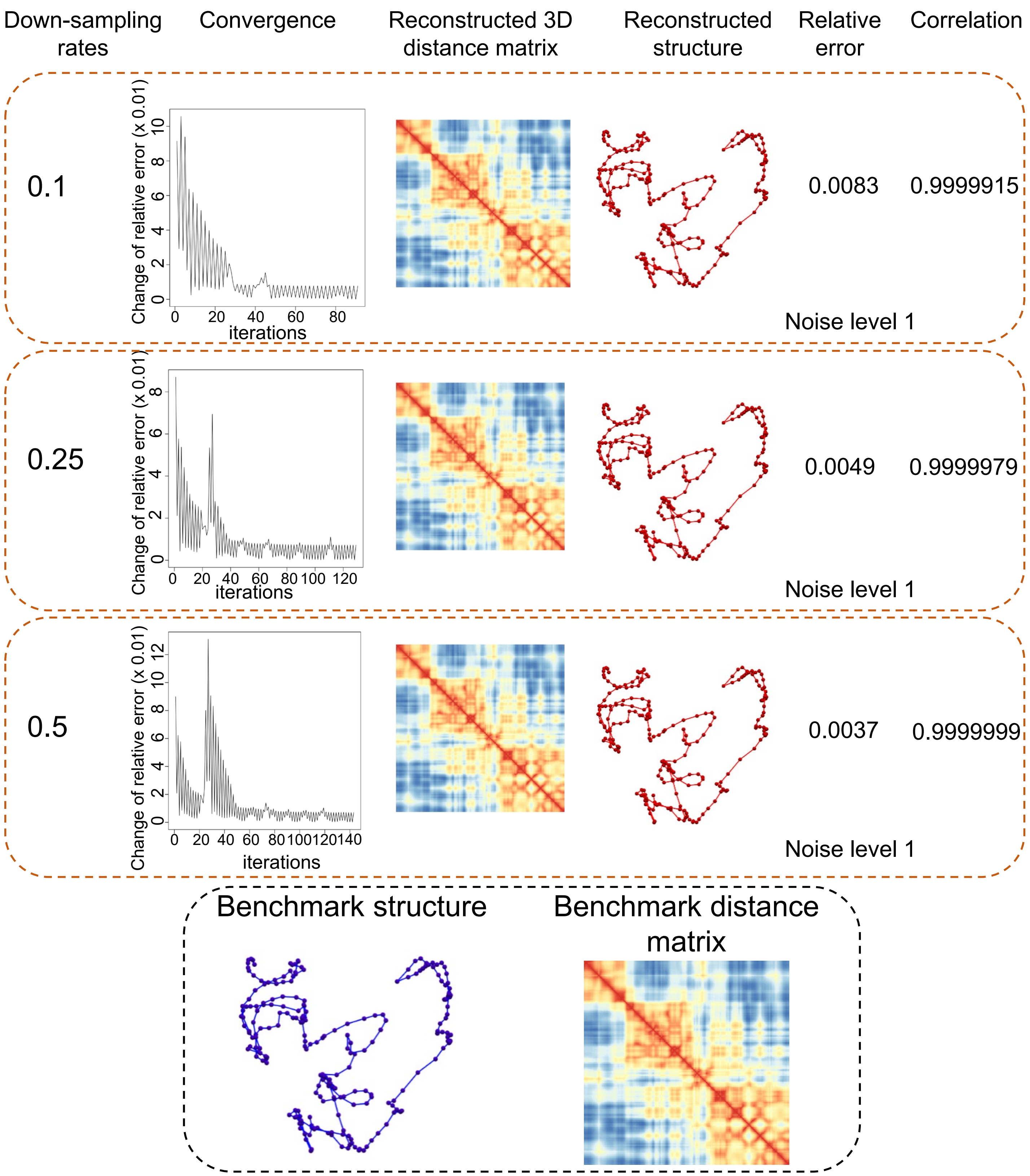

Supplementary Figure 5. Convergence and model performance under different down-sampling rates based on simulated structures. The down-sampled benchmark distance matrices with different down-sampling rates (left column) are mixed with noise and are then used as inputs for FLAMINGO. The convergence is measured by the change of relative error of 3D coordinates between two consecutive iterations (middle-left column). The results of reconstructions are visualized using reconstructed distance matrices (middle column) and reconstructed 3D genome structures (middle-right column). The model performance is quantified by the relative errors and the correlations between the reconstructed and the benchmark distance matrices (right two columns). The benchmark structure and distance matrix are included for comparisons (bottom).

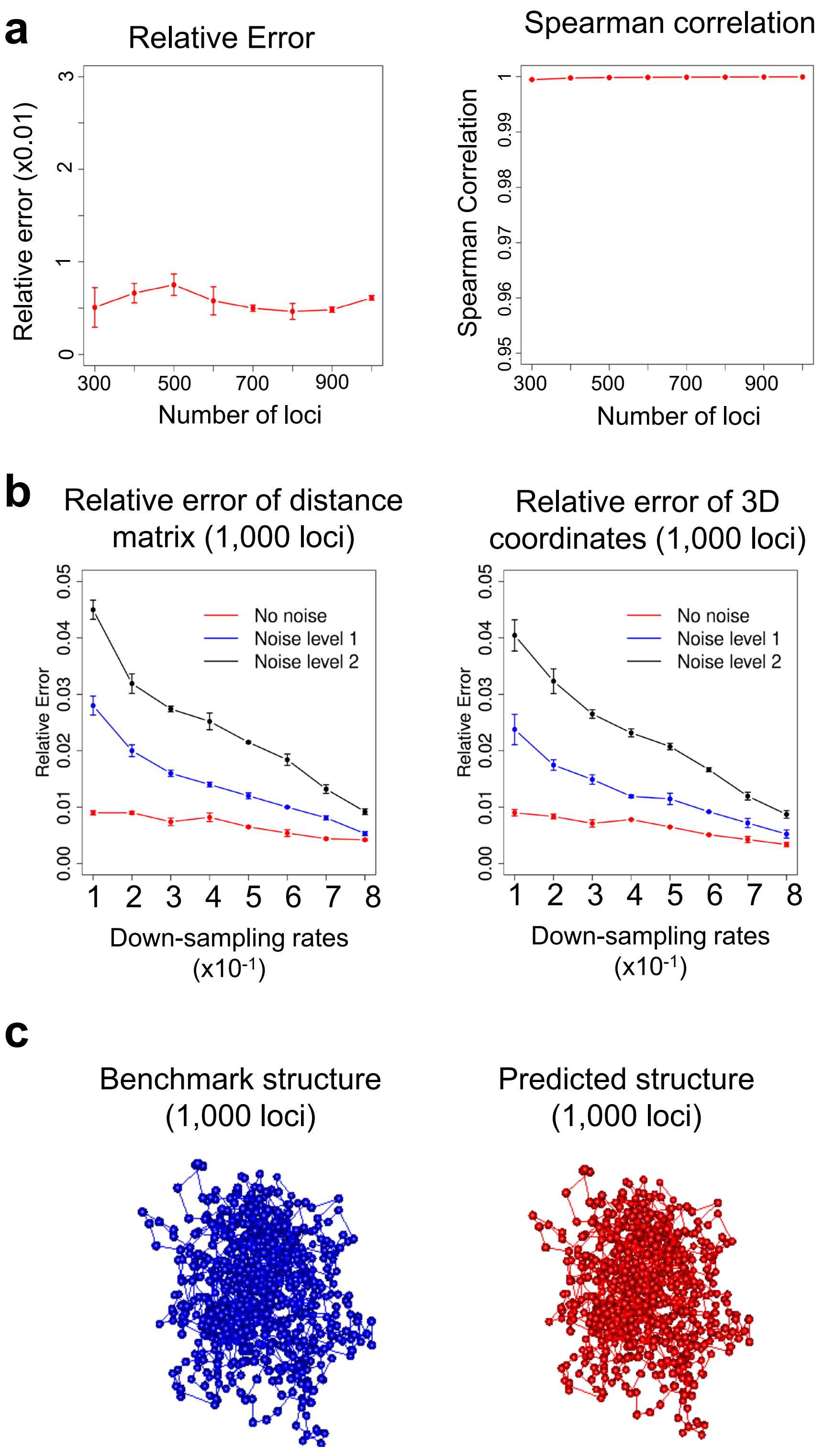

Supplementary Figure 6. Model performance under different number of loci and down-sampling rates based on simulated structures. (**a**) The benchmark structures and distance matrices with different number of loci are generated and are then used as inputs for FLAMINGO. The model performance is evaluated by the relative error (left) and the Spearman correlation (right) between the reconstructed and the benchmark distance matrices. The dots represent the average (mean) relative errors and the average (mean) correlations across *n*=10 random structures and the error bars represent the standard deviations. (**b**) The benchmark structure with 1,000 loci are mixed with different levels of noise. The noisy distance matrices are further down-sampled with different down-sampling rates and used as inputs for FLAMINGO. The relative error based on the distance matrices (left) and 3D coordinates (right) are used to quantify the model performance. The dots represent the average (mean) relative errors and the average (mean) correlations across *n*=10 random structures and the error bars represent the standard deviations. The benchmark structure and predicted structure are shown in (**c**). Source data are provided as a Source Data file.

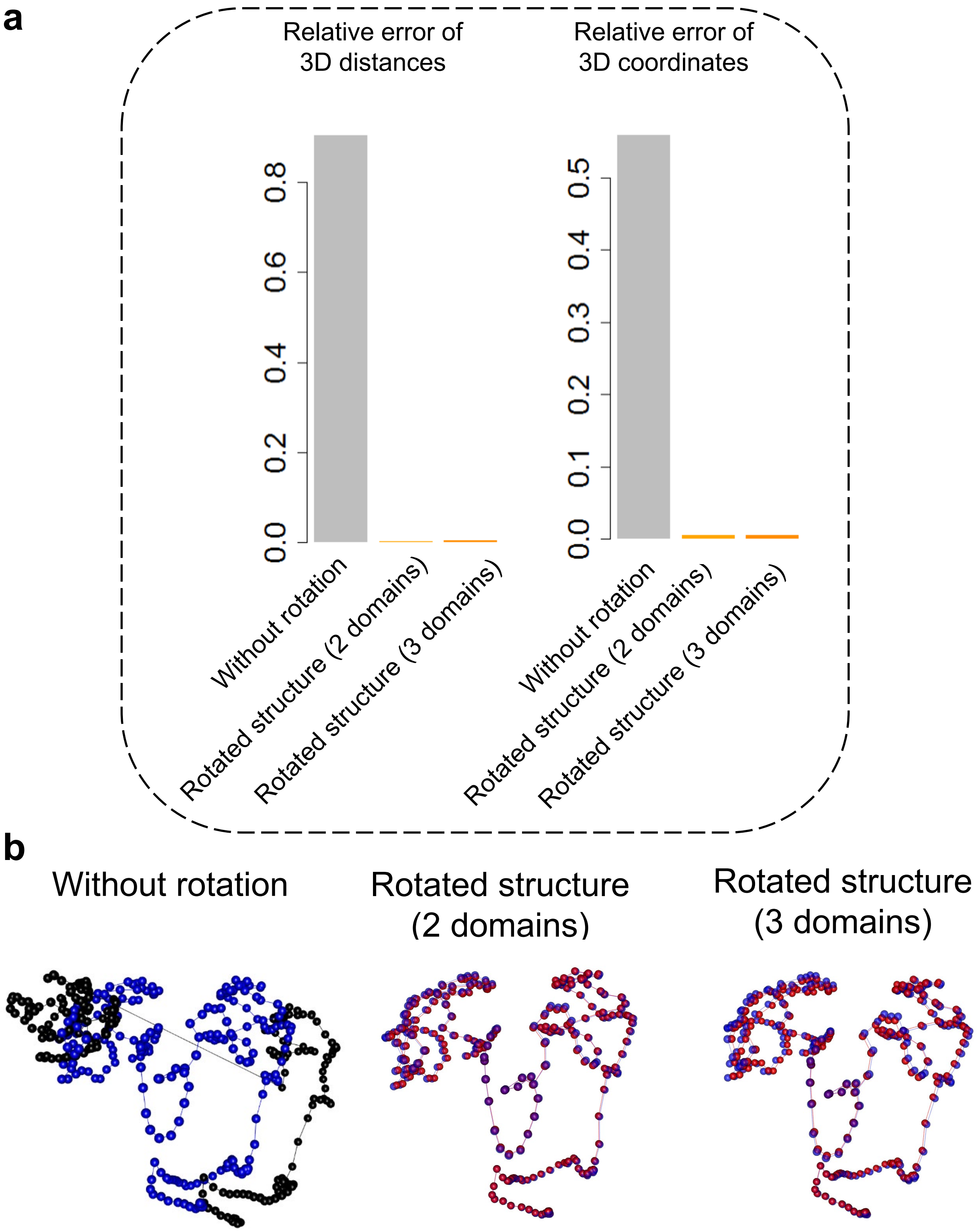

Supplementary Figure 7. Validation of the assembly algorithm based on simulations. (**a**) The benchmark structure is divided into two or three domains. The reconstructed structure is generated using the hierarchical prediction and assembly strategy of FLAMINGO. The performance is evaluated based on the relative errors of the 3D distance matrix (left) and 3D coordinates (right). The assembly algorithm is robust to different numbers of domains and different ways of domain segmentation. (**b**) The visualization of predicted structures under different settings (black: structure without rotation; red: rotated structure; blue: benchmark structure). Source data are provided as a Source Data file.

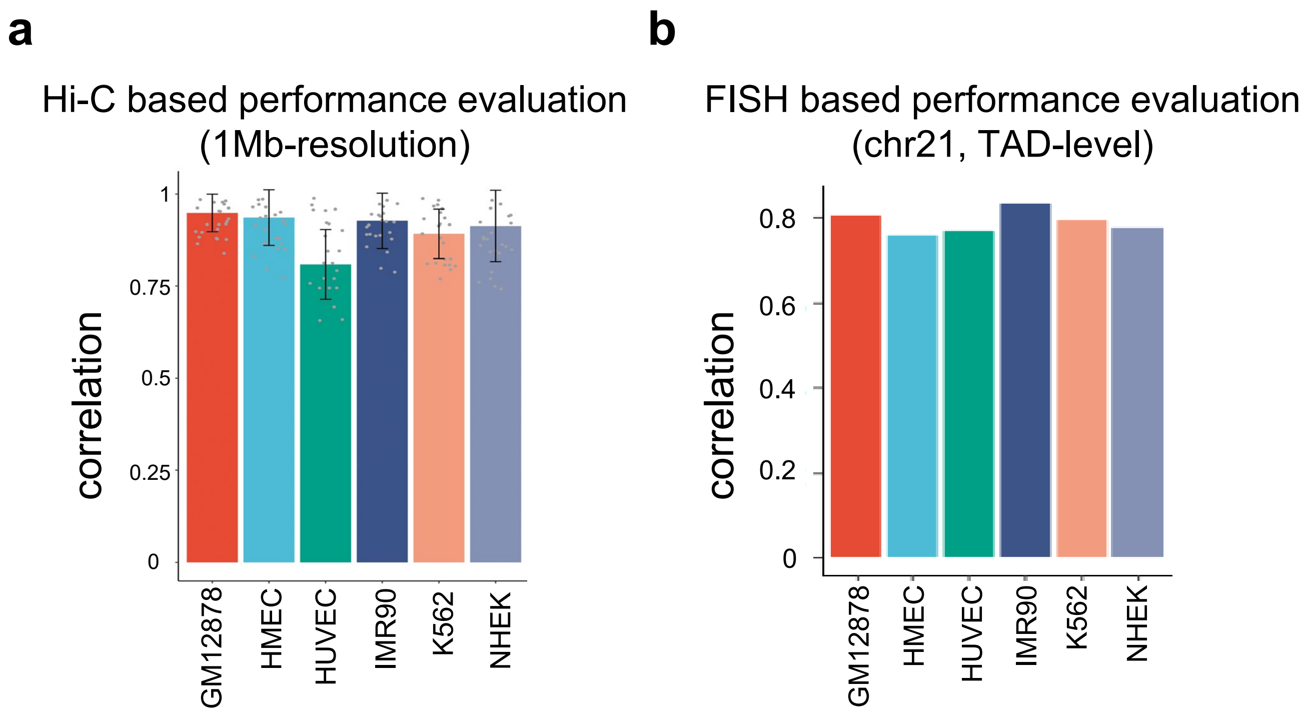

Supplementary Figure 8. Performance validation using low-resolution Hi-C data and FISH data across six cell-types. (**a**) The Spearman correlations between reconstructed and observed distance matrices (from Hi-C) at 1Mb-resolution. The bar plot shows the average (mean) correlation across *n*=23 chromosomes and the error bars represent standard deviation across *n*=23 chromosomes. (**b**) The Spearman correlations between reconstructed and observed distance matrices (from FISH data) for chr21 at TAD-level. For each TAD, the predicted coordinates of TAD-centers are used as the TAD-level 3D coordinates. Source data are provided as a Source Data file.

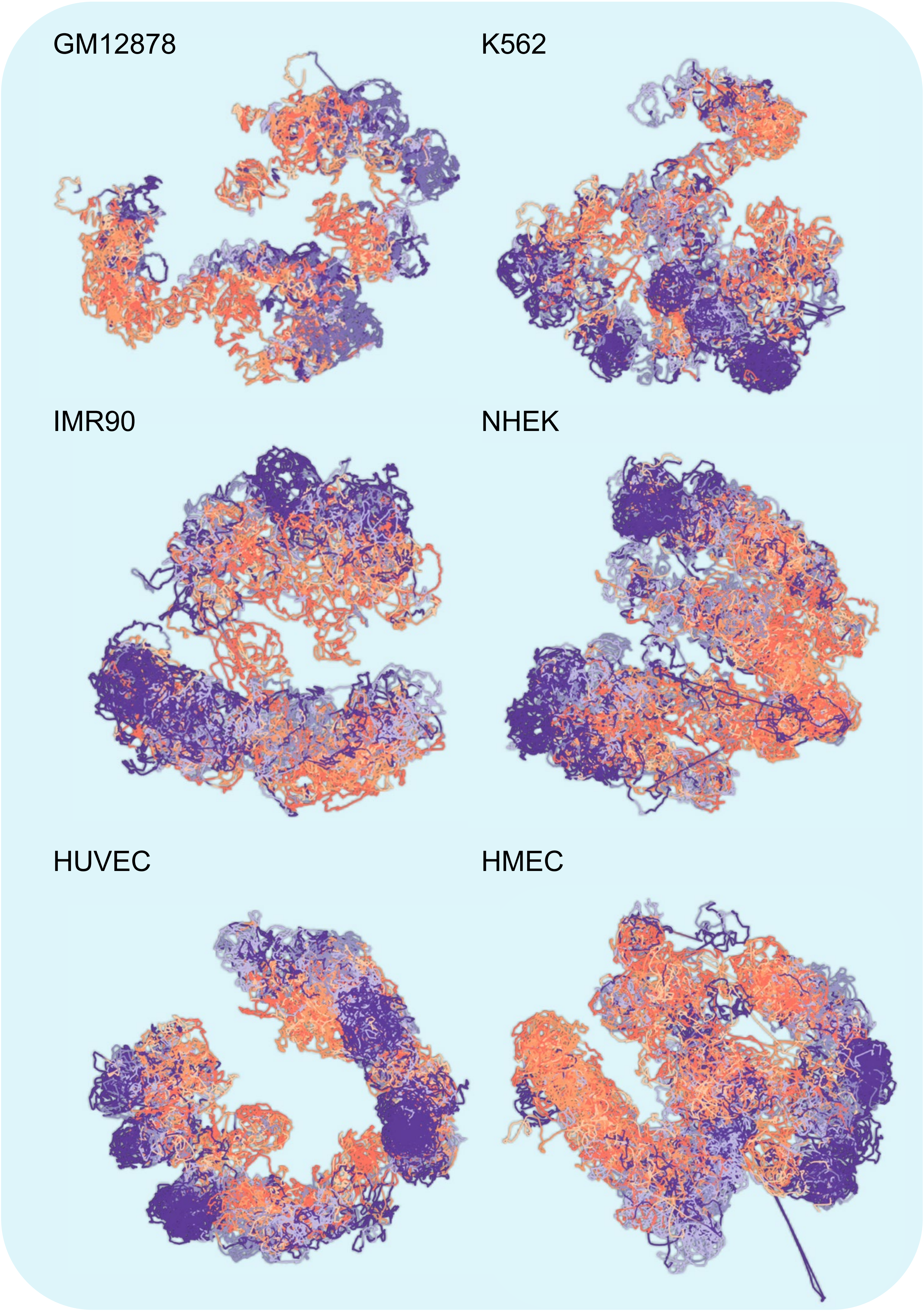

Supplementary Figure 9. Predicted 3D structures of chromosome 1 by FLAMINGO in six cell-types at 5kb-resolution based on Hi-C data.

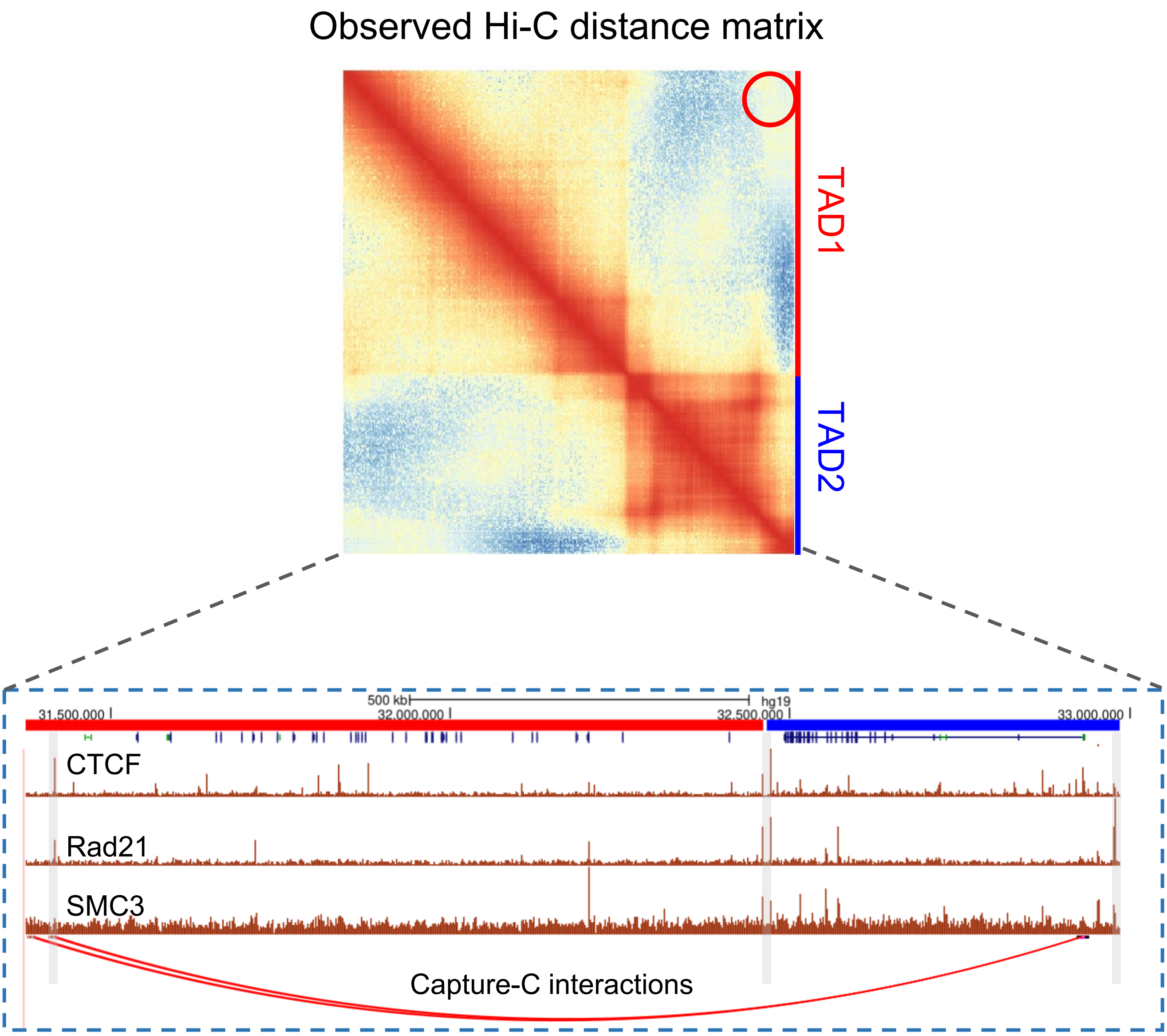

Supplementary Figure 10. The observed 5kb-resolution distance matrix from Hi-C in the genomic region, chr21:31,375,000-32,985,000 which contains two annotated TADs. Vague long-range inter-TAD chromatin interactions are supported by the ChIP-seq signals of CTCF, Rad21 and SMC3. The Capture-C interactions from *Jung et al. (Nature Genetics, 2019)* further support the long-range inter-TAD chromatin interactions predicted by FLAMINGO.

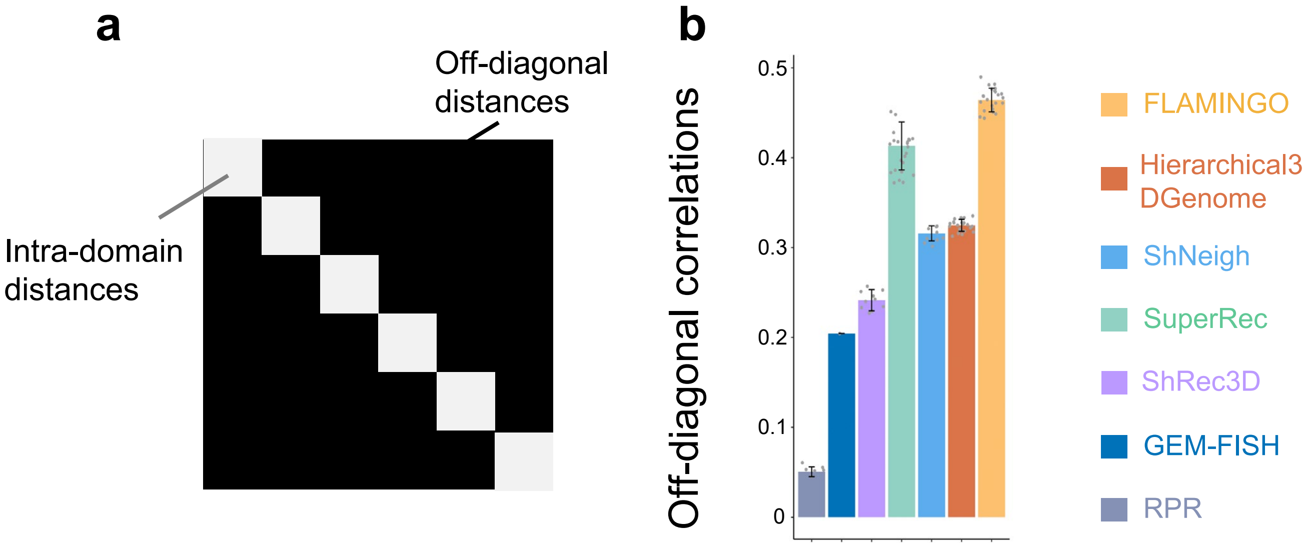

Supplementary Figure 11. Performance comparisons in GM12878 based only on the off-diagonal pairwise distances at 5kb-resolution. (**a**) The off-diagonal distances (black) are determined by excluding the intra-domain distances (grey) from all pairwise distances. (**b**) Comparison of Spearman correlations of the off-diagonal distances from the observed Hi-C data and reconstructed structures. The bar plot shows the average (mean) correlations across all completed chromosomes and the error bars show the standard deviations of correlations (*n*=23 for FLAMINGO, Hierarchical3Dgenome and SuperRec; *n*=10 for ShRec3D; *n*=9 for ShNeigh; *n*=6 for RPR. GEM-FISH does not have error bars because it can only complete the prediction for the chromosome 21). Source data are provided as a Source Data file.

a

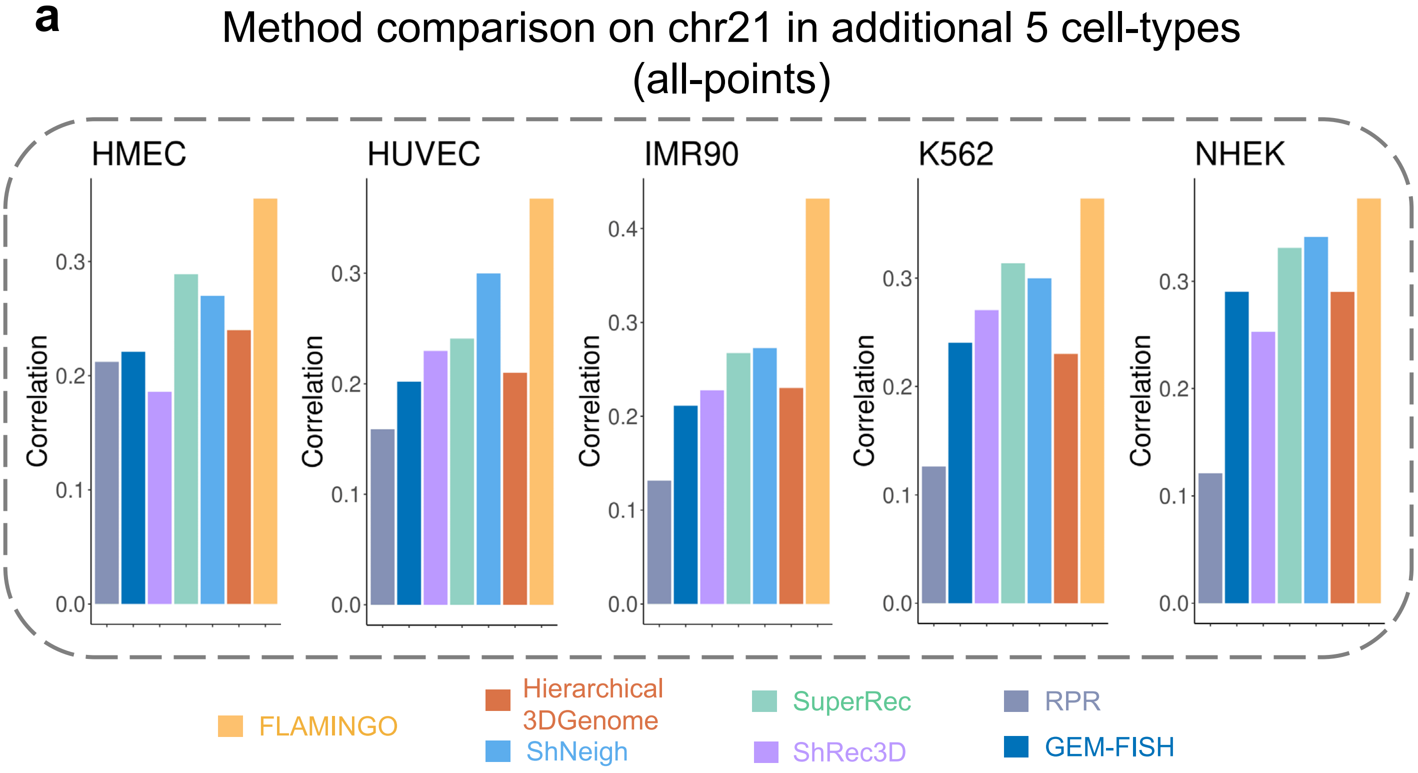

b

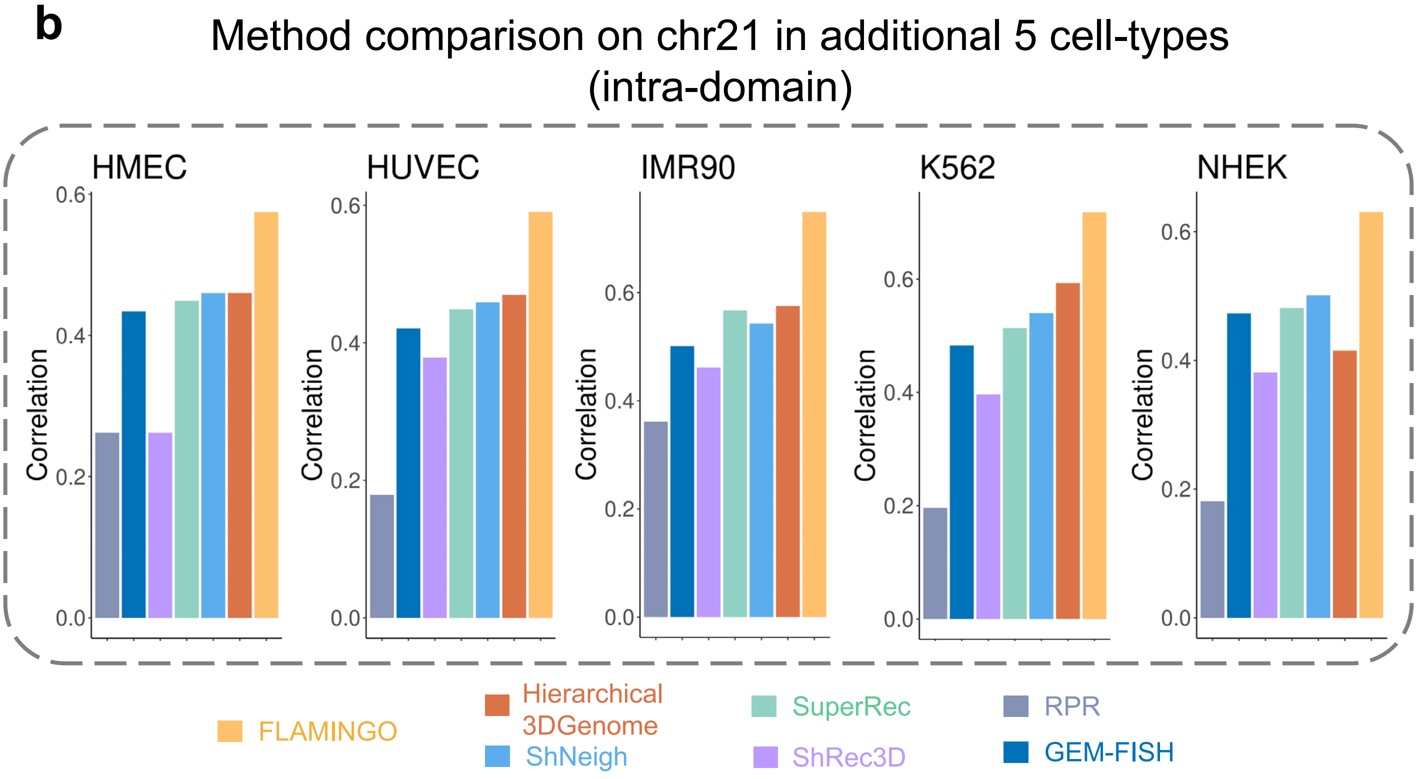

Supplementary Figure 12. Performance comparisons in the additional five cell-types (HMEC, K562, IMR90, NHEK and HUVEC). The model performance is quantified by correlations between predicted and observed distances for all DNA fragments, *i.e.* all-points correlations (**a**); and for DNA fragments within the same domains, *i.e.* intra-domain correlations (**b**). Source data are provided as a Source Data file.

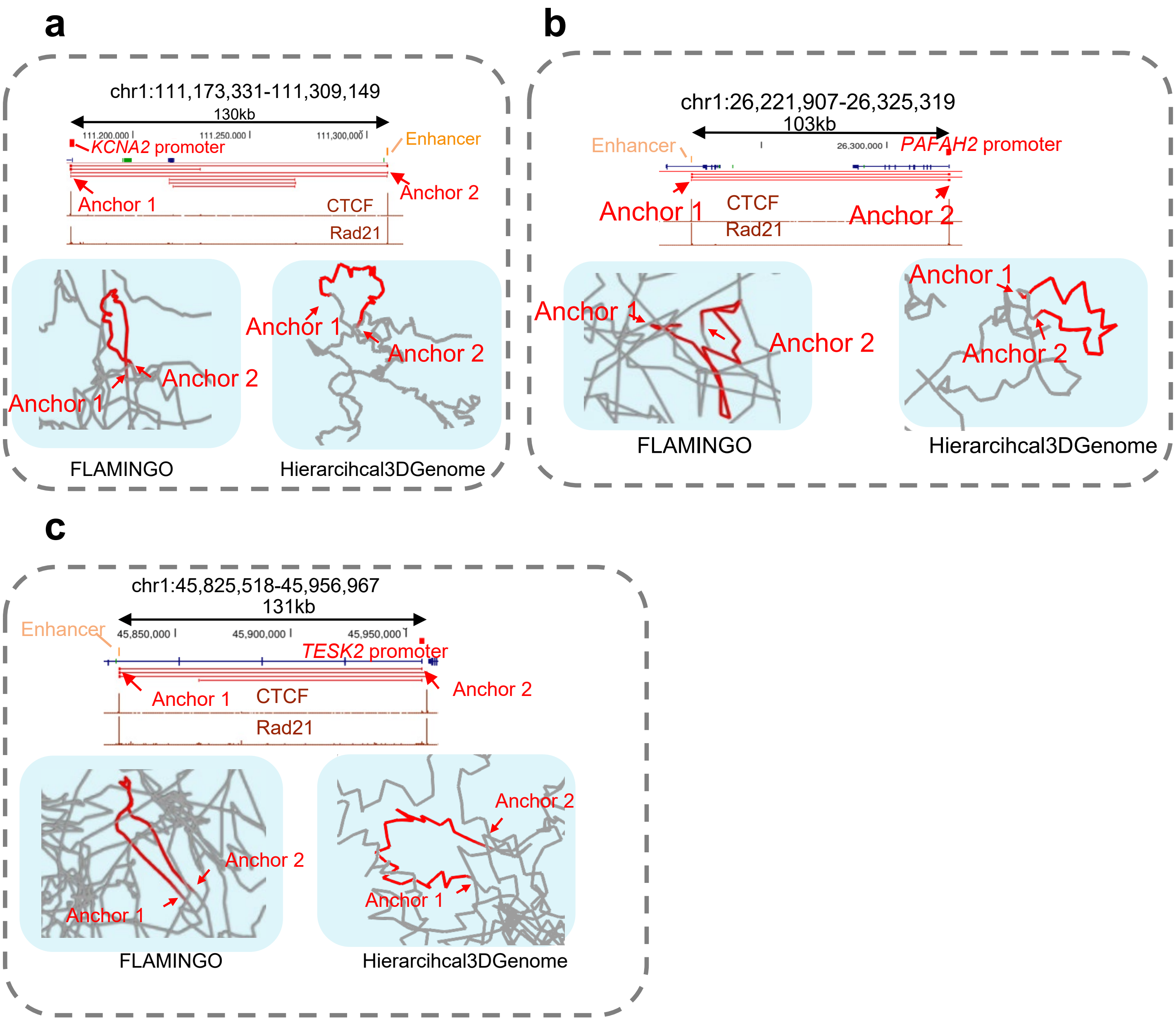

Supplementary Figure 13. Examples of 3D chromatin loops reconstructed by FLAMINGO and Hierarchical3DGenome.The 3D distances are normalized across different methods for fair comparison. (**a**) Long-range ChIA-PET interactions (red links) link a distal enhancer (orange) to the KCNA2 promoter (red). Both anchors are bound by CTCF and Rad21. In 3D space, FLAMINGO predicts a short 3D distance (0.083) between the two anchors, despite the long 1D genomic distance (130kb). In comparison, Hierarchical3DGenome predicts the 3D distance to be 0.128 between the two anchors. (**b**) Long-range ChIA-PET interactions (red links) link a distal enhancer (orange) to the PAFAH2 promoter (red). Both anchors are bound by CTCF and Rad21. In 3D space, FLAMINGO predicts a short 3D distance (0.103) between the two anchors, despite the long 1D genomic distance (103kb). In comparison, Hierarchical3DGenome predicts the 3D distance to be 0.132 between the two anchors. (**c**) Long-range ChIA-PET interactions linking a distal enhancer to the TESK2 promoter. Both anchors are bound by CTCF and Rad21. FLAMINGO predicts a short 3D distance (0.092) between the two anchors, although the 1D genomic distance is 131kb. In comparison, Hierarchical3DGenome predicts the 3D distance to be 0.143.

a The runtime and memory usage of FLAMINGO across all 23 chromosomes

Chromosome ID	Chromosome size (5kb fragments)	Run time (mins)	Memory (GB)	Fraction of missing data
1	44,027	42	2.2	0.945
2	44,872	41.2	2.3	0.937
3	38,583	38.6	1.8	0.925
4	35,105	39.6	1.7	0.934
5	32,167	37.5	1.6	0.925
6	31,977	38.4	1.6	0.92
7	30,352	38.1	1.5	0.919
8	26,246	37.6	1.3	0.908
9	17,608	35.7	1.2	0.933
10	25,363	39.5	1.3	0.9
11	25,826	40	1.2	0.895
12	25,390	38	1.2	0.904
13	15,304	33.9	1	0.917
14	13,845	35.2	1	0.907
15	12,284	33.4	1	0.911
16	13,126	31.6	1	0.889
17	14,170	31.6	1	0.866
18	14,427	30.1	1	0.852
19	10,791	31.6	1	0.815
20	11,585	28.2	1	0.815
21	6,896	26.7	1	0.875
22	6,684	27.2	1	0.875
X	28,818	39.1	1.8	0.911

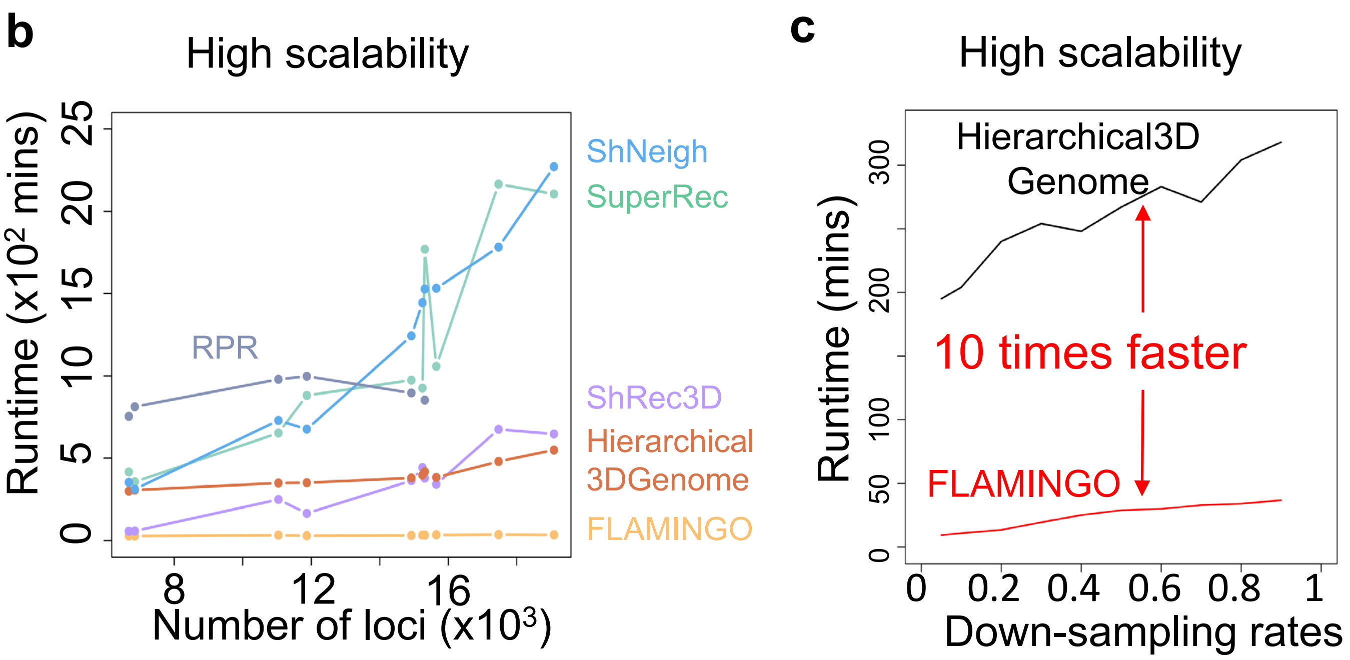

Supplementary Figure 14. High scalability of FLAMINGO. (**a**) Summary of the applications of FLAMINGO in 23 chromosomes. The runtime and the memory usage are tested in high-performance computing environment with AMD EPYC processor using 25 CPUs. The large fractions of missing data in Hi-C is also summarized for 5kb-resolution distance matrices. (**b**) Comparison of the computational scalability by measuring the runtime (the y-axis) as a function of different numbers of genomic loci (the x-axis). (**c**) Comparison of the runtimes (y-axis) of FLAMINGO and Hierarchical3DGenome on chromosome 21 with different down-sampling rates (x-axis).

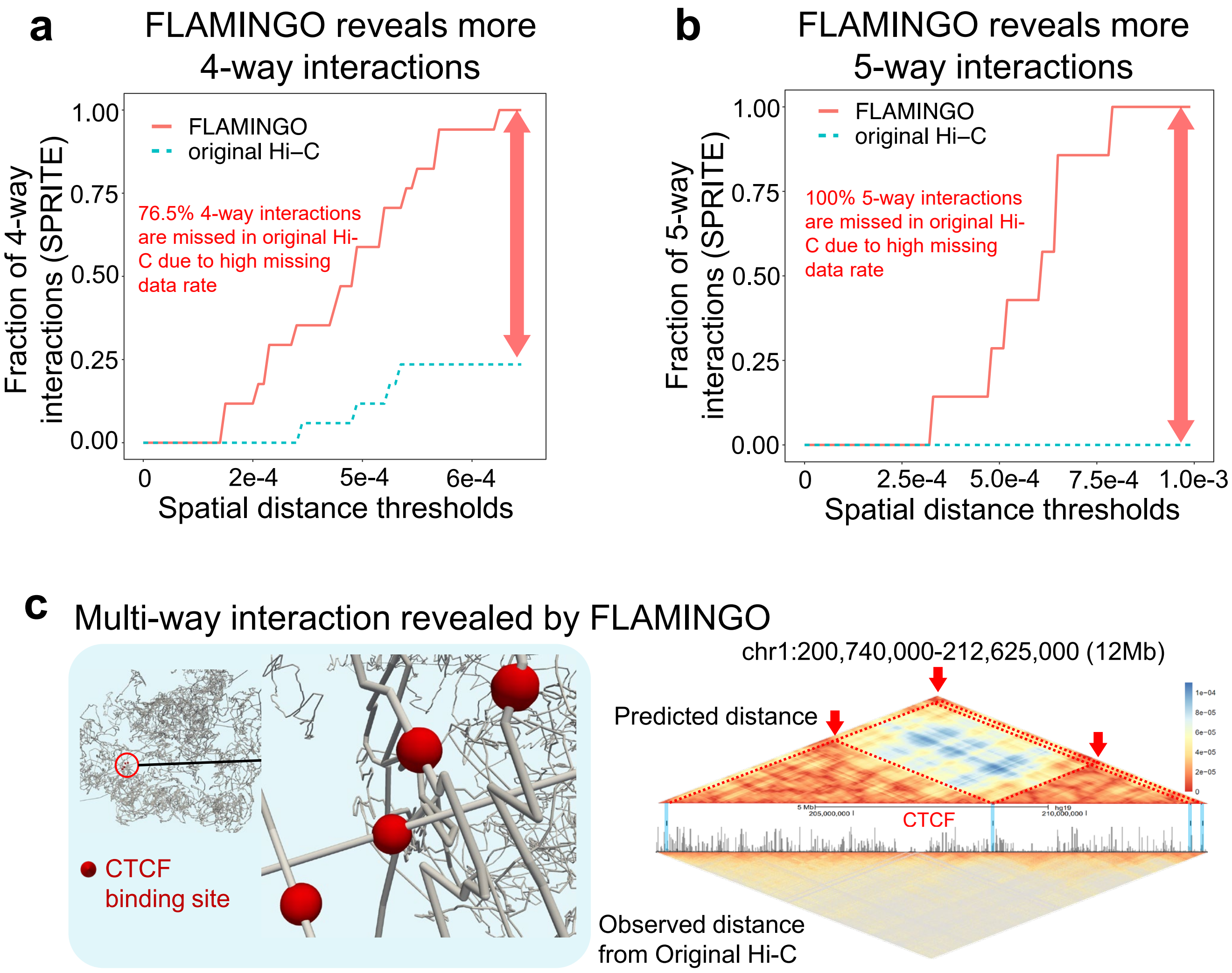

Supplementary Figure 15. (**a**),(**b**) The predicted 3D structure by FLAMINGO reveals more 4-way and 5-way chromatin interactions across a range of different distance thresholds on chr21, compared to using Hi-C contact map derived distance matrix. The spatial distances predicted by FLAMINGO and the Hi-C contact map derived distances are normalized for fair comparisons. Besides, 76.5% SPRITE significant 4-way interactions and 100% SPRITE significant 5-way interactions can not be found using Hi-C contact map derived distance matrix, due to the high rate of missing data. (**c**) One example of a candidate 4-way chromatin interaction across a 12Mb genomic region identified by FLAMINGO. All four interacting anchors (i.e. genomic regions) are bound by CTCF. The distance matrix derived from Hi-C contact map demonstrates almost no signal of long-range chromatin interactions for this region. Source data are provided as a Source Data file.

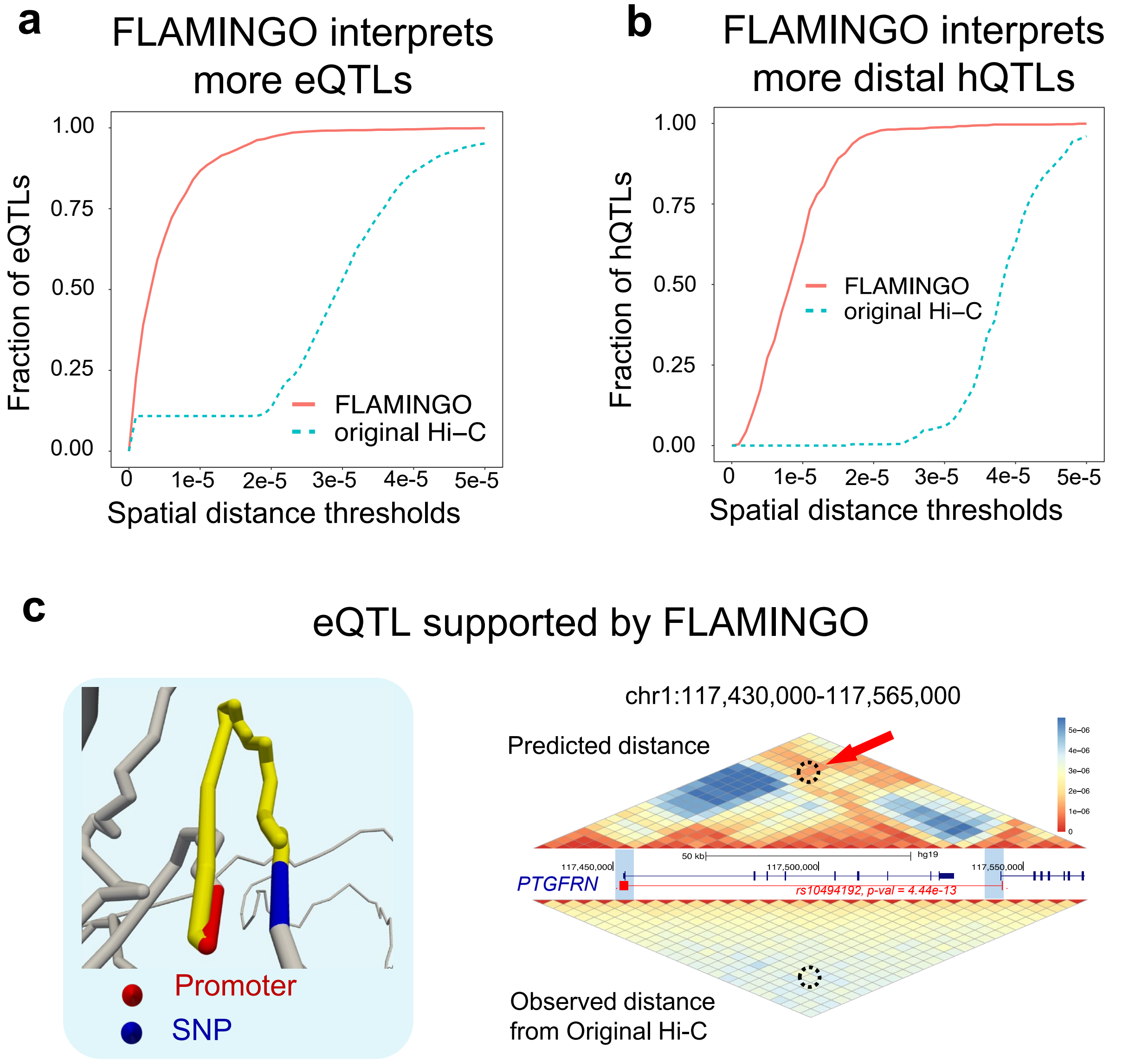

Supplementary Figure 16. (**a**) FLAMINGO interprets more eQTLs across different distance thresholds on chr1, compared to using Hi-C contact map derived distance matrix. Distances are normalized for fair comparisons. (**b**) FLAMINGO interprets more distal hQTLs across different distance thresholds on chr1, compared to using Hi-C contact map derived distance matrix. (**c**) One example of an eQTL supported by FLAMINGO. The SNP rs10494192 (blue) and the promoter of PTGFRN (red) are placed in close 3D proximity, which can not be found based on the distance matrix from the original Hi-C contact map. Source data are provided as a Source Data file.

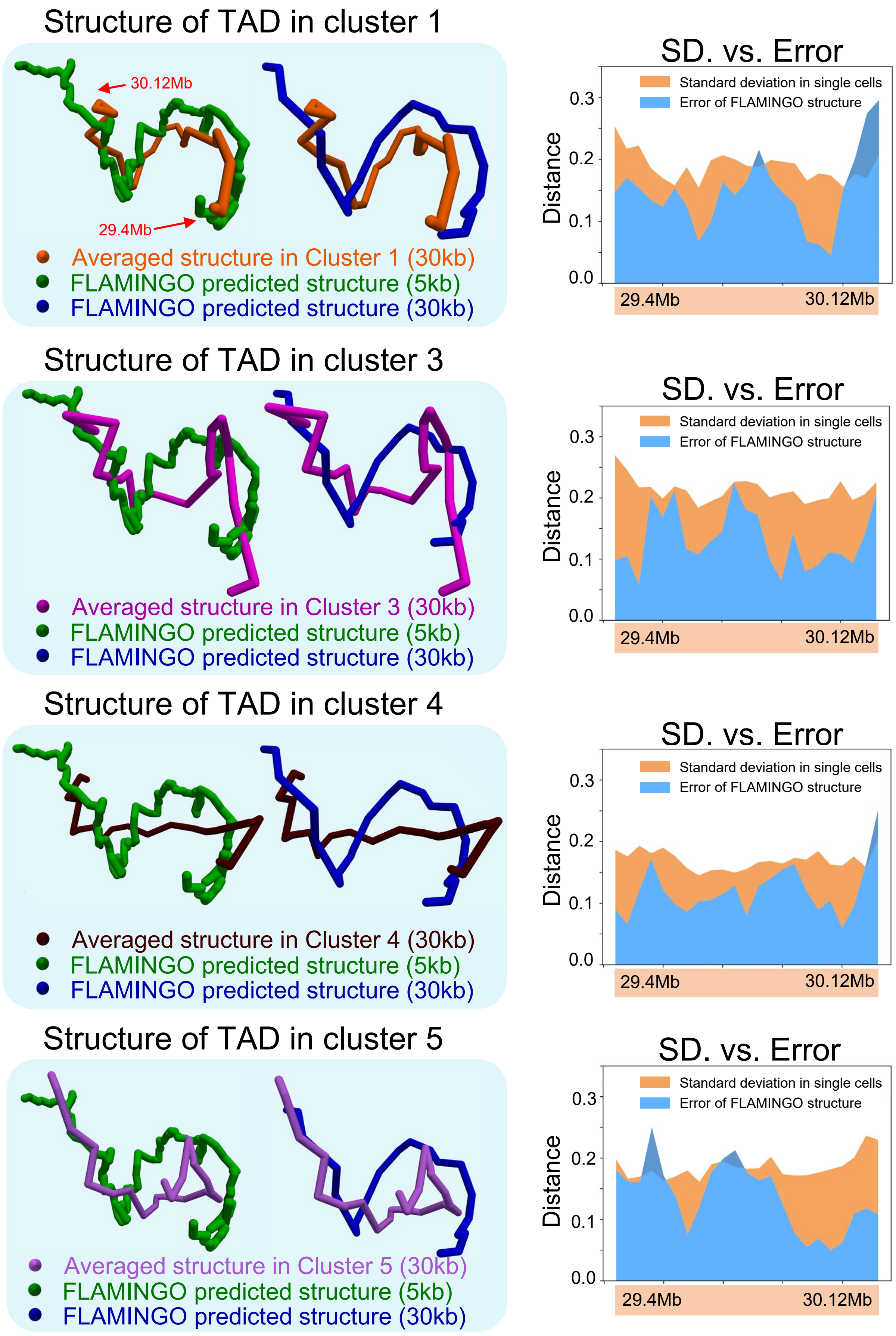

Supplementary Figure 17. Compare the predicted structure by FLAMINGO with the other four single-cell clusters in K562. For each cluster, the cluster-specific average structures (30kb-resolution) are aligned with the 5kb- and 30kb-resolution predicted structures from FLAMINGO. Right: The differences between the predicted structure and the cluster-specific average structures (blue) are smaller than the intrinsic standard deviations among single cells within each cluster (orange). Source data are provided as a Source Data file.

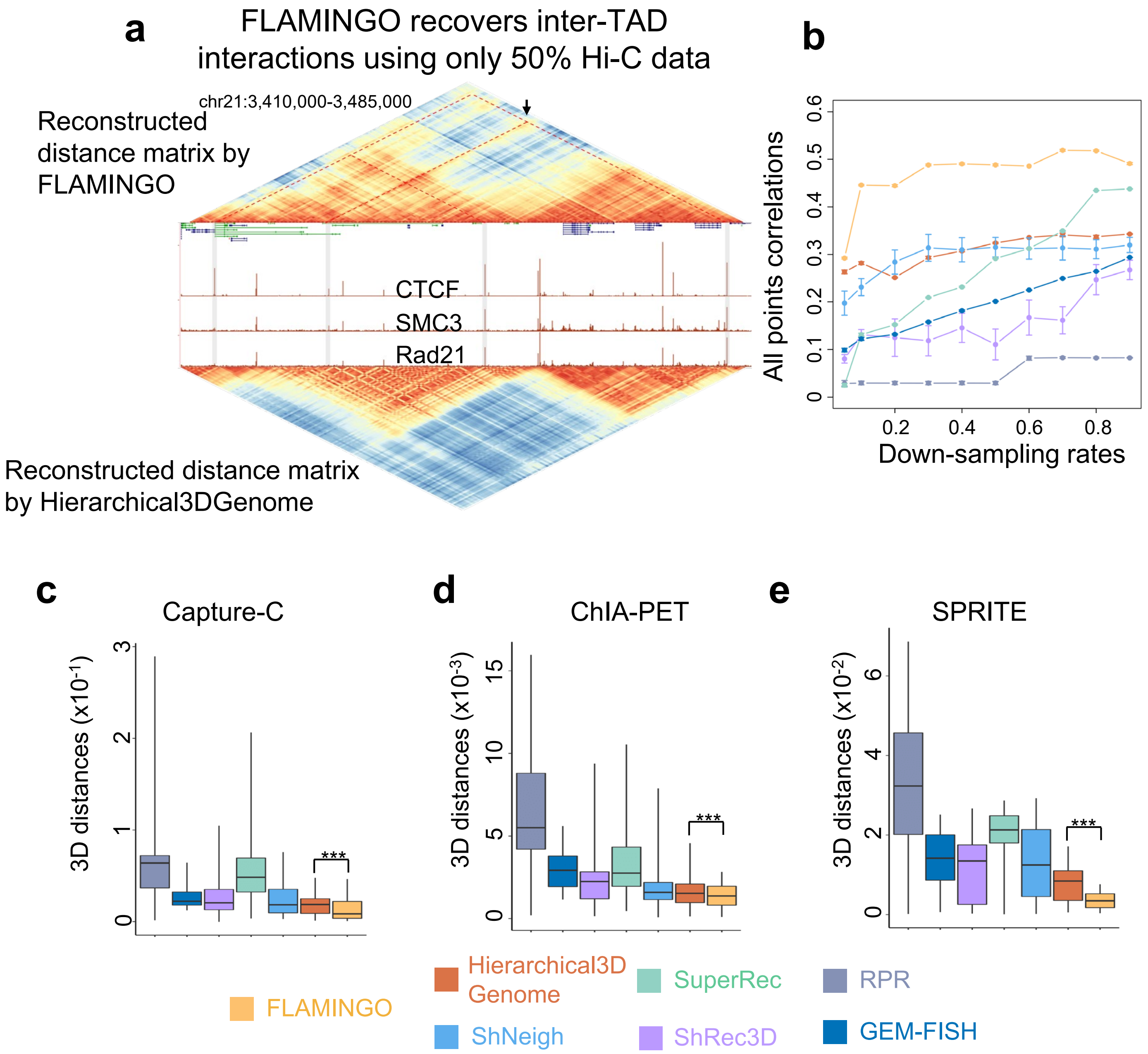

Supplementary Figure 18. FLAMINGO robustly reconstructs the high-resolution 3D structures using a small fraction of observed Hi-C data. (**a**) FLAMINGO identifies the long-range chromatin interactions (highlighted by black arrows) based on down-sampled input matrix from Hi-C data (down-sampling rate=50%) and recovers the high-resolution complete distance matrix (top), while Hierarchical3DGenome fails to discover these high-resolution structural features. The identified long-range chromatin interactions are supported by the bindings of CTCF and cohesin. (**b**) Comparison of the model performance (all-points correlation) under different down-sampling rates (x-axis) on chromosome 21. The dots show the average (mean) correlations based on *n*=10 independently down-sampled input matrices and the error bars represent the standard deviations of correlations. (**c-e**) Comparison of the distances between anchors of chromatin interactions profiled by Capture-C (*p-value* = 1.33×10^{-19}, *n*=3,692) (**c**), ChIA-PET (*p-value* = 5.87×10^{-17}, *n*=214) (**d**) and SPRITE (*p-value* = 7.53×10^{-23}, *n*=871) (**e**) across all methods (one-sided Mann-Whitney test). The input data of all methods are down-sampled by 50%. The 3D structures of different methods are normalized for fair comparison. The center lines of boxplots show the median, the upper and lower box limits show the 25th and 75th percentiles respectively. The whiskers extend up to 1.5 times the interquartile range away from the limits of the boxes. Outliers outside this range were removed from the figure. Source data are provided as a Source Data file.

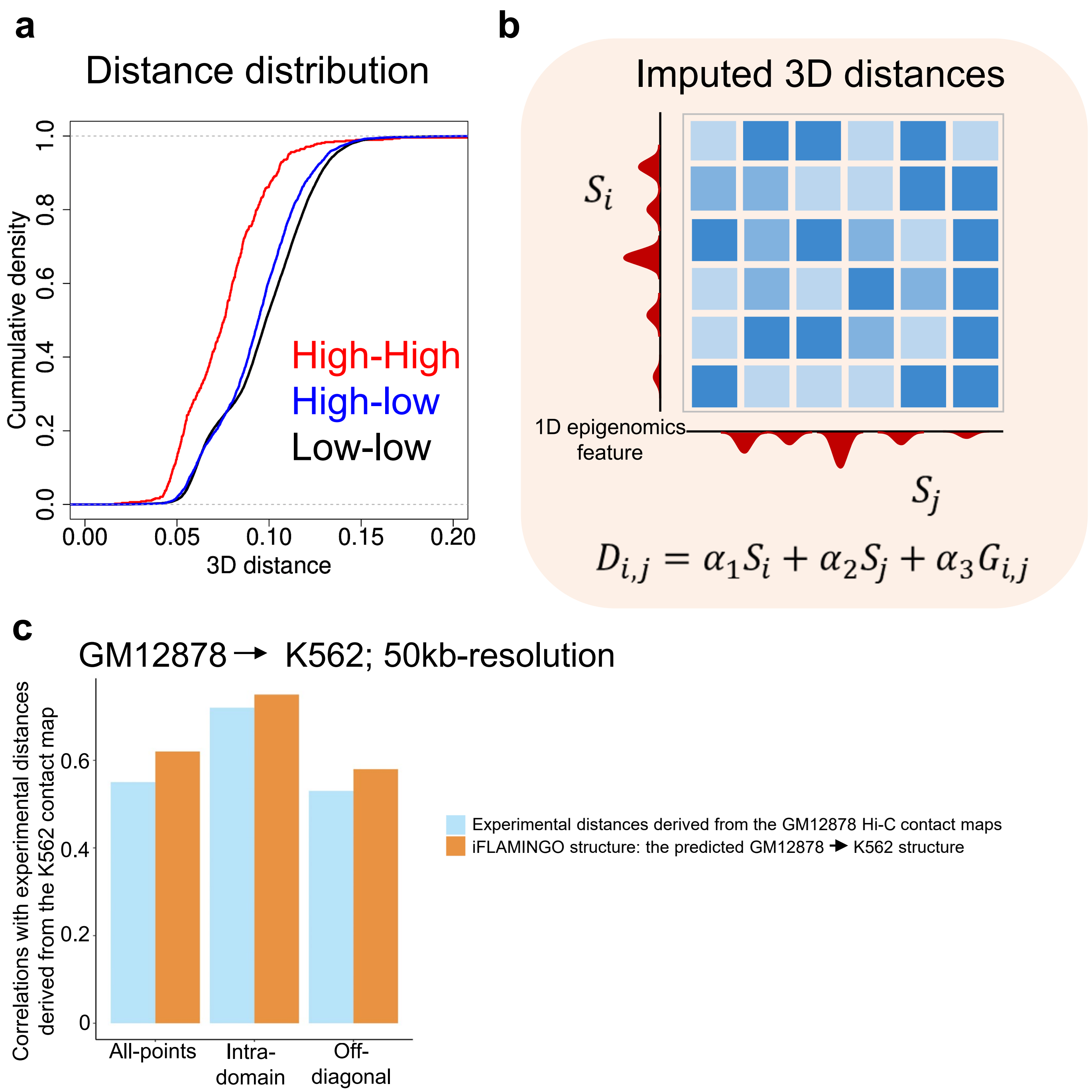

Supplementary Figure 19. The imputation of 3D distances based on 1D epigenomics data in iFLAMINGO. (**a**) DNA fragments are considered to have high DNase-seq signals if the value is ranked in the top 5% among all fragments. If both DNA fragments show high DNase-seq signals, they tend to show shorter 3D distances (*p-value* $< 2.2 \times 10^{-16}$, one-sided Kolmogorov-Smirnov test) (**b**) Overview of the 3D distance imputation. The imputed distance between fragment i and fragment j ($D_{i,j}$) is estimated with the linear model. For each fragment pair (i and j), epigenomic signals (e.g. DNase-seq) of two DNA fragments (S_i and S_j) and 1D genomic distance ($G_{i,j}$) are used as independent variables. The regression coefficients are estimated from the observed distances based on Hi-C data. (**c**) Cross-cell type prediction of the 3D chromatin structure in K562 from GM12878 at 50kb-resolution. The Hi-C data in GM12878 and DNase-seq data in K562 are used to reconstruct the 3D chromatin structures in K562 (the predicted GM12878->K562 structure) by iFLAMINGO. The correlations between the predicted distances from the GM12878->K562 structure and the experimentally-derived distances from the K562 Hi-C contact map are calculated (orange). As comparison, the correlations based on experimental distances derived from Hi-C contact maps in GM12878 and K562 are calculated (light blue).

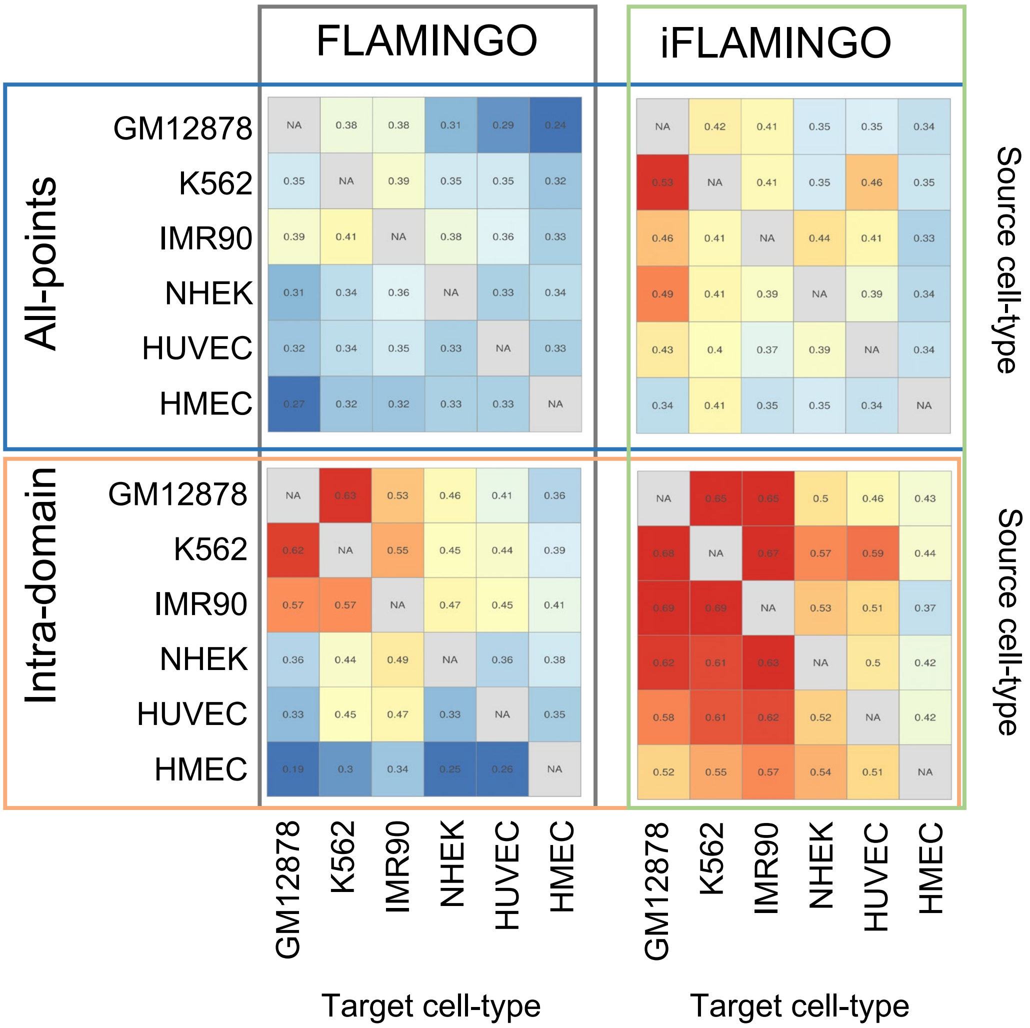

Supplementary Figure 20. Performance of cross cell-type predictions using iFLAMINGO. In total, 30 source-target pairs of cell-types are included in the evaluation. The model performances are quantified by all-points correlations and intra-domain correlations to target cell-type structures. Rows represent the source cell-types and columns represent the target cell-types. The performance of iFLAMINGO, which integrates DNase-seq from target cell-types, (right column) is compared with FLAMINGO, which only uses Hi-C from source cell-types (left column).

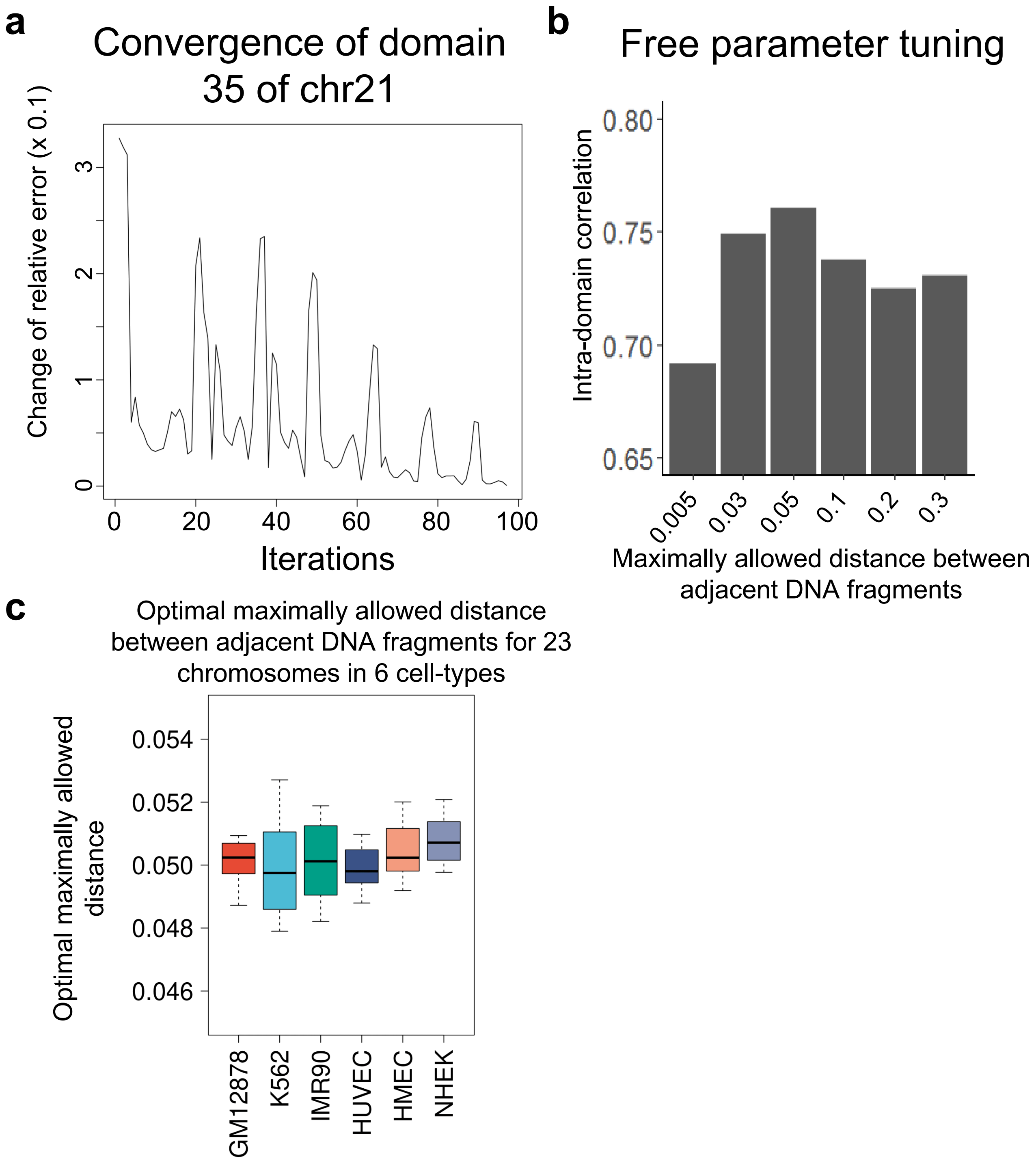

Supplementary Figure 21. Convergence and parameter tuning of FLAMINGO based on the real Hi-C data. (**a**) Example of the fast convergence of FLAMINGO on domain 35 of chromosome 21. The convergence is quantified by the change of relative error of 3D coordinates between consecutive iterations (y-axis). (**b**) The model performance based on different values of the maximally allowed distance between adjacent DNA fragments. The performance is quantified by intra-domain correlations. The value=0.05 is selected as the default value for FLAMINGO. (**c**) The optimal maximally allowed distance between adjacent DNA fragments for all 23 chromosomes in 6 cell-types (*n*=23). 100 values of the maximally allowed distance ranging from 0.04 to 0.06 are tested respectively. The center lines of boxplots show the median, the upper and lower box limits show the 25th and 75th percentiles respectively. The whiskers extend up to 1.5 times the interquartile range away from the limits of the boxes. Outliers outside this range were removed from the figure. Source data are provided as a Source Data file.

Supplementary Note 1

Additional data collection and processing

The annotations of chromatin compartments and TAD domains in the six cell-types are collected from Rao et al.[1] (GSE63525). The DNase-seq data in the six cell-types are collected from the ENCODE and Roadmap Epigenome Consortia[2, 3] (https://egg2.wustl.edu/roadmap/web_portal/processed_data.html). The FISH data of chromosome 21 is collected from Wang et al.[4]. The significant ChIA-PET interactions in GM12878 are collected from the ENCODE project[2] (GSE127053/ENCSR981FNA). The significant ChIA-PET interactions in K562 are collected from GEO[5] (GSE33664). The Capture-C data in GM12878 is collected from Jung et al.[6] (GSE86189). To remove the low-quality Capture-C interactions, we filter the Capture-C interactions using the p-value threshold of 0.01 as suggested by the paper. The SPRITE data is collected from Quinodoz et al.[7] (GSE114242) and significant SPRITE interactions are identified as described in the original paper. The ChIP-seq signal tracks of TFs are collected from the ENCODE project[2] (CTCF: GSM822312/ENCSR000DKV; SMC3: GSM935376/ENCSR000DZP; RAD21: GSM935332/ENCSR000EAB). Promoters are defined as the +/- 1kb region around TSS of genes, based on gene annotation GENCODE V17[8]. The enhancer annotations are collected from the Roadmap Epigenome Consortium[3].

Algorithm to align 3D structures

The predicted 3D structures from different algorithms need to be aligned with each other. To enable direct visual comparisons between predicted structures, we rotate and align the 3D structures for visual comparisons using the following method. Given a 0-centered reference structure ($\mathbf{R}$) and a 0-centered query structure ($\mathbf{Q}$) with n points, the optimal rotation matrix ($\mathbf{r}$) from the query structure to the reference structure is calculated as $\mathbf{U}\mathbf{V}^{\mathrm{T}}$, where $\mathbf{U}$ and $\mathbf{V}$ are calculated from the singular vector decomposition of matrix $\mathbf{Q}^{\mathrm{T}}\mathbf{R}$. The rotated query structure is calculated as $\mathbf{Q}^{\mathbf{r}} = \mathbf{Q}\mathbf{r}$, which optimally aligns with the reference structure.

Computational complexity of FLAMINGO

The overall computation complexity of FLAMINGO is $O(kN^2)$, where k is the down-sampling rates (*i.e.* rates of missing data) and N is the number of loci. To calculate the gradients in Barzilai-Borwein steepest descent strategy, the most computationally intensive step is the operator A and its adjoint A^*, where $A(\mathbf{X}) = f \in R^{|\Omega|*1}, f_i =< \mathbf{X}, \boldsymbol{\omega}_{\alpha_i} >, for\ \alpha_i \in \Omega$. In our setting, the size of Ω is kN^2. It can be shown that the operation is linear: $f_i(\mathbf{X}) = < \mathbf{X}, \boldsymbol{\omega}_{\alpha_i} >= < \mathbf{X}, \mathbf{e}_{\alpha_{i,1},\alpha_{i,1}} + \mathbf{e}_{\alpha_{i,2},\alpha_{i,2}} - \mathbf{e}_{\alpha_{i,1},\alpha_{i,2}} - \mathbf{e}_{\alpha_{i,2},\alpha_{i,1}} >= X_{i,i} + X_{j,j} - X_{i,j} - X_{j,i}$, which yields 4 calculations per α_i and, thus, totally $4kN^2$ calculations. The adjoint of A (A^*) can be calculated as $A^*\mathbf{y} = \sum_i y_i \boldsymbol{\omega}_{\alpha_i}$, which is also a linear operator involving totally $4kN^2$ calculations. All together, the computation complexity of FLAMINGO is in the order of $O(kN^2)$.

Performance validation using FISH data

The FISH data experimentally measures the 3D coordinates of TADs, the typical size of which are hundreds of kilobases. Therefore, the FISH data provides additional evidence to support the predictive accuracy of FLAMINGO. The 5kb DNA fragments located at the center of each TAD are used to represent the predicted 3D coordinates of TADs. The Spearman correlations between the observed structures from the FISH data and the predicted structures by FLAMINGO are calculated to quantify the model performance.

Notes on model comparison

We compare FLAMINGO with seven state-of-the-art algorithms: PASTIS[9], RPR[10], GEM-FISH[11], ShRec3D[12], ShNeigh[13], SuperRec[14] and Hierarchical3DGenome[15]. These algorithms have been shown to perform better than other methods in each category by previous studies. The seven models are tested on the same AMD EPYC processors with the maximum runtime set to be three days and the maximum memory set to be 100 GB. 25 CPUs are allocated for each method if parallel computing is needed.

PASTIS models the 3D genome structure using a probabilistic model. The stand-alone package of PASTIS is collected from GitHub (https://GitHub.com/hiclib/pastis/). The PM2 model is tested, as suggested by the original paper. The maximum number of iterations is set to 500 to guarantee model convergence. Due to scalability issues, PASTIS does not converge in three days.

RPR models the binarized contact maps (1 for observed contacts and 0 for others) using the recurrent plot. The scripts of RPR are collected from the original paper. The default suggested parameters are used. RPR can complete predictions for chromosomes 17-22 within three days.

GEM-FISH employs a manifold learning based model to predict 3D structures based on Hi-C and FISH data. GEM-FISH software is collected from GitHub (https://GitHub.com/ahmedabbas81/GEM-FISH) and tested based on the instructions. GEM-FISH can only complete the predictions for chromosome 21.

ShRec3D is an MDS-based approach to reconstruct 3D genome structures based on the shortest-path distances, which is generated by applying the shortest-path algorithm (*i.e.* the Floyd-Warshall algorithm) on the observed distance matrix from Hi-C. The ShRec3D software is downloaded from GitHub (https://GitHub.com/kpj/ShRec3D). The observed distance matrix is created based on the conversion factor η=0.25 from Hi-C data, which is the same as FLAMINGO. The missing data in the observed distance matrix is filled with 0 as instructed. ShRec3D can complete predictions for chromosomes 13-22 within three days.

ShNeigh is an improved MDS-based method by modeling the 3D proximity of neighboring points. The stand-alone package of ShNeigh is downloaded from Github (https://github.com/fangzhen-li/ShNeigh) and tested following the instruction. ShNeigh can finish the prediction of chromosomes 15-22 and X.

SuperRec reconstructs the 3D genome structures based on an iterative weighted MDS method. The SuperRec software is collected from http://www.cs.cityu.edu.hk/~shuaicli/SuperRec. SuperRec can finish the prediction for all chromosomes within three days.

Hierarchical3DGenome combines the optimization of the Lorentzian function and a hierarchical prediction strategy to predict 3D structures. The software is downloaded from GitHub (https://GitHub.com/BDM-Lab/Hierarchical3DGenome) and tested as instructed. Hierarchical3DGenome can complete predictions for all 23 chromosomes.

To directly compare the predicted 3D distances between DNA fragments from different models, we unify the predicted 3D coordinates as: $\mathbf{P}_{\text{unify}} = \mathbf{P}/||\mathbf{P}||_{\text{F}}$, where $\mathbf{P}$ is the predicted 3D coordinates and $||\mathbf{P}||_F$ is the Frobenius norm of the coordinate matrix. The 3D distances based on the unified coordinates can be directly compared with each other and are used for downstream analyses.

CTCF motif identification

The CTCF motif hits along the human genome are collected from the motif browser[16] using the confidence score threshold of 0.3. The control motifs are filtered out. The directions of CTCF motifs are decided by aligning DNA sequences of motif hits and the CTCF motif using TOMTOM[17].

References

1. Rao, S.S. et al. A 3D map of the human genome at kilobase resolution reveals principles of chromatin looping. *Cell* **159**, 1665-1680 (2014).

2. Consortium, E.P. An integrated encyclopedia of DNA elements in the human genome. *Nature* **489**, 57-74 (2012).
3. Roadmap Epigenomics, C. et al. Integrative analysis of 111 reference human epigenomes. *Nature* **518**, 317-330 (2015).
4. Wang, S. et al. Spatial organization of chromatin domains and compartments in single chromosomes. *Science* **353**, 598-602 (2016).
5. Li, G. et al. Extensive promoter-centered chromatin interactions provide a topological basis for transcription regulation. *Cell* **148**, 84-98 (2012).
6. Jung, I. et al. A compendium of promoter-centered long-range chromatin interactions in the human genome. *Nat Genet* **51**, 1442-1449 (2019).
7. Quinodoz, S.A. et al. Higher-Order Inter-chromosomal Hubs Shape 3D Genome Organization in the Nucleus. *Cell* **174**, 744-757 e724 (2018).
8. Frankish, A. et al. GENCODE reference annotation for the human and mouse genomes. *Nucleic Acids Res* **47**, D766-D773 (2019).
9. Varoquaux, N., Ay, F., Noble, W.S. & Vert, J.P. A statistical approach for inferring the 3D structure of the genome. *Bioinformatics* **30**, i26-33 (2014).
10. Hirata, Y., Oda, A., Ohta, K. & Aihara, K. Three-dimensional reconstruction of single-cell chromosome structure using recurrence plots. *Scientific Reports* **6**, 34982 (2016).
11. Abbas, A. et al. Integrating Hi-C and FISH data for modeling of the 3D organization of chromosomes. *Nat Commun* **10**, 2049 (2019).
12. Lesne, A., Riposo, J., Roger, P., Cournac, A. & Mozziconacci, J. 3D genome reconstruction from chromosomal contacts. *Nat Methods* **11**, 1141-1143 (2014).
13. Li, F.-Z. et al. Chromatin 3D structure reconstruction with consideration of adjacency relationship among genomic loci. *BMC Bioinformatics* **21**, 272 (2020).
14. Zhang, Y., Liu, W., Lin, Y., Ng, Y.K. & Li, S. Large-scale 3D chromatin reconstruction from chromosomal contacts. *BMC Genomics* **20**, 186 (2019).
15. Trieu, T., Oluwadare, O. & Cheng, J. Hierarchical Reconstruction of High-Resolution 3D Models of Large Chromosomes. *Sci Rep* **9**, 4971 (2019).

16. Kheradpour, P. & Kellis, M. Systematic discovery and characterization of regulatory motifs in ENCODE TF binding experiments. *Nucleic Acids Res* **42**, 2976-2987 (2014).

17. Gupta, S., Stamatoyannopoulos, J.A., Bailey, T.L. & Noble, W.S. Quantifying similarity between motifs. *Genome Biol* **8**, R24 (2007).
