# Supplementary material for: Reconstruct high-resolution 3D genome structures for diverse cell-types using FLAMINGO
Source: Nat Commun. 2022 May 12;13:2645. doi: 10.1038/s41467-022-30270-2 (PMC9098643; doi:10.1038/s41467-022-30270-2)
Supplement: Supplementary file 2 — Reporting Summary [file 41467_2022_30270_MOESM2_ESM.pdf]

nature research

Corresponding author(s): *Jianrong Wang*

Last updated by author(s): *04/14/2022*

Reporting Summary

Nature Research wishes to improve the reproducibility of the work that we publish. This form provides structure for consistency and transparency in reporting. For further information on Nature Research policies, see our Editorial Policies and the Editorial Policy Checklist.

Statistics

For all statistical analyses, confirm that the following items are present in the figure legend, table legend, main text, or Methods section.

n/a	Confirmed	
☐	☒	The exact sample size (n) for each experimental group/condition, given as a discrete number and unit of measurement
☒	☐	A statement on whether measurements were taken from distinct samples or whether the same sample was measured repeatedly
☐	☒	The statistical test(s) used AND whether they are one- or two-sided<br>*Only common tests should be described solely by name; describe more complex techniques in the Methods section.*
☒	☐	A description of all covariates tested
☒	☐	A description of any assumptions or corrections, such as tests of normality and adjustment for multiple comparisons
☐	☒	A full description of the statistical parameters including central tendency (e.g. means) or other basic estimates (e.g. regression coefficient) AND variation (e.g. standard deviation) or associated estimates of uncertainty (e.g. confidence intervals)
☐	☒	For null hypothesis testing, the test statistic (e.g. F, t, r) with confidence intervals, effect sizes, degrees of freedom and P value noted<br>*Give P values as exact values whenever suitable.*
☒	☐	For Bayesian analysis, information on the choice of priors and Markov chain Monte Carlo settings
☒	☐	For hierarchical and complex designs, identification of the appropriate level for tests and full reporting of outcomes
☐	☒	Estimates of effect sizes (e.g. Cohen's d, Pearson's r), indicating how they were calculated

Our web collection on statistics for biologists contains articles on many of the points above.

Software and code

Policy information about availability of computer code

Data collection	No software was used for data collection.
Data analysis	The code of FLAMINGO is available at https://github.com/wangjr03/FLAMINGO.<br><br>Genome wide epigenetics analysis: BEDTools(v2.27.1).<br><br>Model implementation related:<br>R (V3.5.1): Matrix (v1.3-2), mgcv(v1.8-34), parallel(v3.6.2), MASS(v7.3-53.1), strawr(v0.0.9), data.table(v1.14.0).<br><br>Visualization:<br>R packages: rgl(v0.105.22), ggplot2 (v3.1.0), RColorBrewer (v1.1.1-2), ggsci (v2.9).<br>Paraview(v5.9.0)<br>Latest version of the UCSC genome browser(as of 03/31/2022).<br><br>Method comparison:<br>We compared the performance of FLAMINGO with ShRec3D (https://github.com/jbmorlot/ShRec-Exented commit version 09b1fbb), RPR (provided as the supplementary information in the original paper link), ShNeigh (https://github.com/fangzhen-li/ShNeigh commit version 79a2176), SuperRec (provided at https://www.cs.cityu.edu.hk/~shuaicli/SuperRec/) and GEM-FISH (https://github.com/ahmedabbas81/GEM-FISH commit version e83fdb4). We use the Tomtom tool (version 5.3.2) for the motif discovery analysis.

For manuscripts utilizing custom algorithms or software that are central to the research but not yet described in published literature, software must be made available to editors and reviewers. We strongly encourage code deposition in a community repository (e.g. GitHub). See the Nature Research guidelines for submitting code & software for further information.

Data

Policy information about availability of data

All manuscripts must include a data availability statement. This statement should provide the following information, where applicable:

- Accession codes, unique identifiers, or web links for publicly available datasets
- A list of figures that have associated raw data
- A description of any restrictions on data availability

Hi-C data, along with the annotations of chromatin compartments and TADs, are available under GSE63525 [https://www.ncbi.nlm.nih.gov/geo/query/acc.cgi?acc=GSE63525].
Capture-C data are available under GSE86189 [https://www.ncbi.nlm.nih.gov/geo/query/acc.cgi?acc=GSE86189].
ChIA-PET data in K562 are available under GSE33664 [https://www.ncbi.nlm.nih.gov/geo/query/acc.cgi?acc=GSE33664] and ChIA-PET data in GM12878 are available under GSE127053 [https://www.ncbi.nlm.nih.gov/geo/query/acc.cgi?acc=GSE127053].
SPRITE data are available under GSE114242 [https://www.ncbi.nlm.nih.gov/geo/query/acc.cgi?acc=GSE114242].
DNase-seq data are available from ENCODE and Roadmap consortia (https://egg2.wustl.edu/roadmap/web_portal/processed_data.html).
ChIP-seq datasets are available from the GEO database (CTCF: GSM822312 [https://www.ncbi.nlm.nih.gov/geo/query/acc.cgi?acc=GSM1002651]; SMC3: GSM935376 [https://www.ncbi.nlm.nih.gov/geo/query/acc.cgi?acc=GSM935376]; Rad21: GSM935332 [https://www.ncbi.nlm.nih.gov/geo/query/acc.cgi?acc=GSM935332]).
The gene annotation is available at GENCODE (https://www.gencodegenes.org/human/release_17.html).
The eQTL datasets are available from the supplementary data of Battle et al, the MuTHER consortia (http://www.muther.ac.uk/Data.html), the Geuvadis project (https://www.ebi.ac.uk/arrayexpress/experiments/E-GEUV-3/) and the GTEx consortium (https://storage.googleapis.com/gtex_analysis_v7/single_tissue_eqtl_data/GTEx_Analysis_v7_eQTL.tar.gz). The hQTL dataset is available at https://www.zaugg.embl.de/data-and-tools/distal-chromatin-qtls/. Single-cell chromatin tracing data is downloaded from https://github.com/BogdanBintu/ChromatinImaging.
The data of simulations, reconstructed chromosome structures, and cross cell-type predictions generated in this study are available in FLAMINGO GitHub repository: https://github.com/wangjr03/FLAMINGO.
Source data are provided with this paper.
Associated data for Figures 2-8 , Supplementary Figures 1-2, Supplementary Figures 6-8, Supplementary Figures 11-12, Supplementary Figures 16-18 and Supplementary Figure 21 are provided as the source data.

Field-specific reporting

Please select the one below that is the best fit for your research. If you are not sure, read the appropriate sections before making your selection.

- [x] Life sciences
- [ ] Behavioural & social sciences
- [ ] Ecological, evolutionary & environmental sciences

For a reference copy of the document with all sections, see nature.com/documents/nr-reporting-summary-flat.pdf

Life sciences study design

All studies must disclose on these points even when the disclosure is negative.

Sample size	We evaluated FLAMINGO using Hi-C contact maps for 23 chromosomes of the human genome in 6 different cell-types (available at GSE63525), 3 orthogonal chromatin interaction datasets (ChIA-PET, Capture-C and SPRITE) and 5 QTL datasets (eQTL data from Battle et al. the MuTHER consortia, the Geuvadis project and GTEx consortium;hQTL data from Grubert et al. See Data availability). These high-quality datasets are selected because they cover a broad diverse range of cell-types and technologies at high-resolution. These datasets were generated by independent analyses with different sample sizes, different technologies and different sequencing depth. The robust performance based on these datasets supports the generalizability and significance of FLAMINGO.
Data exclusions	No data were excluded from analyses.
Replication	No new experimental datasets were generated. Computational replications were performed in data analysis and performance evaluation. The evaluations based on the simulated datasets were repeated 10 times and all attempts at replication were successful. In down-sampling analysis, the down-sampling procedure was repeated 10 times to confirm the robustness of the model and all attempts at replication were successful.
Randomization	Randomization in experiments was not relevant for this study as we do not generate new experimental data. In simulation and data analysis, the input datasets were randomly selected in all computational replicates.
Blinding	Since we report the FLAMINGO algorithm as the main finding and there is no experiment in this study, the experimental blinding is not relevant. For computational predictions, blinding was not relevant since FLAMINGO did not use the information of labels to make predictions. For algorithm performance comparisons, all algorithms were evaluated independently and in parallel.

Reporting for specific materials, systems and methods

We require information from authors about some types of materials, experimental systems and methods used in many studies. Here, indicate whether each material, system or method listed is relevant to your study. If you are not sure if a list item applies to your research, read the appropriate section before selecting a response.

Materials & experimental systems

n/a	Involved in the study
☒	☐ Antibodies
☒	☐ Eukaryotic cell lines
☒	☐ Palaeontology and archaeology
☒	☐ Animals and other organisms
☒	☐ Human research participants
☒	☐ Clinical data
☒	☐ Dual use research of concern

Methods

n/a	Involved in the study
☒	☐ ChIP-seq
☒	☐ Flow cytometry
☒	☐ MRI-based neuroimaging
